# Synthesis of Bioconjugate Sesterterpenoids with Phospholipids and Polyunsaturated Fatty Acids

**DOI:** 10.3390/molecules21010047

**Published:** 2015-12-30

**Authors:** Ana Gil-Mesón, Alejandro M. Roncero, Ignacio E. Tobal, Pilar Basabe, David Díez, Faustino Mollinedo, Isidro S. Marcos

**Affiliations:** 1 Departamento de Química Orgánica, Universidad de Salamanca, Plaza de los Caídos 1-5, 37008 Salamanca, Spain; anagm@usal.es (A.G.-M.); alexmaron@usal.es (A.M.R.); ignaciotobal@usal.es (I.E.T.); pbb@usal.es (P.B.); ddm@usal.es (D.D.); 2 Instituto de Biología Molecular y Celular del Cáncer, Centro de Investigación del Cáncer, CSIC-Universidad de Salamanca, Campus Miguel de Unamuno, 37007 Salamanca, Spain; fmollin@usal.es; 3 Laboratory of Cell Death and Cancer Therapy, Department of Cellular and Molecular Medicine, Centro de Investigaciones Biológicas, CSIC, C/Ramiro de Maeztu 9, 28040 Madrid, Spain

**Keywords:** antitumoural, bioconjugates, ether lipidics, edelfosine, sesterterpenolides, PUFAs

## Abstract

A series of sesterterpenoid bioconjugates with phospholipids and polyunsaturated fatty acids (PUFAs) have been synthesized for biological activity testing as antiproliferative agents in several cancer cell lines. Different substitution analogues of the original lipidic ether edelfosine (1-*O*-octadecyl-2-*O*-methyl-*rac*-glycero-3-phosphocholine) are obtained varying the sesterterpenoid in position 1 or 2 of the glycerol or a phosphocholine or PUFA unit in position 3. Simple bioconjugates of sesterterpenoids and eicosapentaenoic acid (EPA) have been obtained too. All synthetic derivatives were tested against the human tumour cell lines HeLa (cervix) and MCF-7 (breast). Some compounds showed good IC_50_ (0.3 and 0.2 μM) values against these cell lines.

## 1. Introduction

There is a growing interest in medicinal chemistry in the synthesis of bioconjugate compounds [1,2,3,4,5]. Bioconjugate molecules have been described as antitumour agents and as analgesics, showing a synergistic effect due to conjugation. Most known bioconjugates are oligonucleotides [6] with lipids, aminoacids with hydrophilic or lipophilic vitamins [7], lipids with sugars [8], that have a synergistic effect due to the conjugation.

Bioconjugates made by direct esterification of paclitaxel (Taxol^®^) with polyunsaturated fatty acids (PUFAs) give good antitumour therapy results as the docosahexaenoic acid (DHA)-paclitaxel bioconjugate is less toxic and stable enough in plasma to have a slow release at the tumour [9,10]. Some of the most studied bioconjugates are alkylglycerol derivatives with different biological active molecules [11,12,13,14,15,16,17,18,19,20]. In many cases, these hybrids are considered prodrugs [10,11].

**Figure 1:**
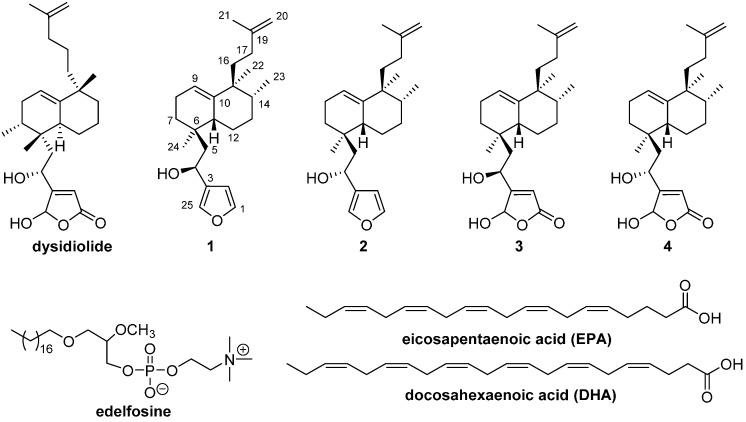
Dysidiolide, edelfosine, PUFAs and sesterterpenoid compounds used for bioconjugation.

In this work, we report the synthesis and biological evaluation of several biological active sesterterpenes derived from dysidiolide [21,22,23,24] (Figure 1) and bioconjugated with phospholipids such as the synthetic ether lipid edelfosine (1-*O*-octadecyl-2-*O*-methyl-*rac*-glycero-3-phosphocholine) [25,26,27,28,29,30,31] and PUFAs [32,33,34,35,36,37,38,39,40,41,42,43], which separately show antitumour activity and together might have a synergistic effect. Edelfosine is most widely studied antitumoural alkyl lipidic ether as it inhibits the cell growth of several tumour cell lines [26,44,45].

The terpenoids used in this work as starting materials for the bioconjugate synthesis were the bioactive nor-sesterterpenoid **3** and **4** analogues of dysidiolide [21,22,23,24], (Figure 1) and the furosesterterpene intermediates **1** and **2**. These compounds have been synthesized previously by our group, starting with *ent*-halimic acid [46], and have a considerable antitumour activity, similar to that of dysidiolide, showing cellular proliferation inhibition (IC_50_ ≈ 4.8–5.4 μM) on several solid tumour cellular lines and leukemia [46].

## 2. Results and Discussion

**Figure 2:**
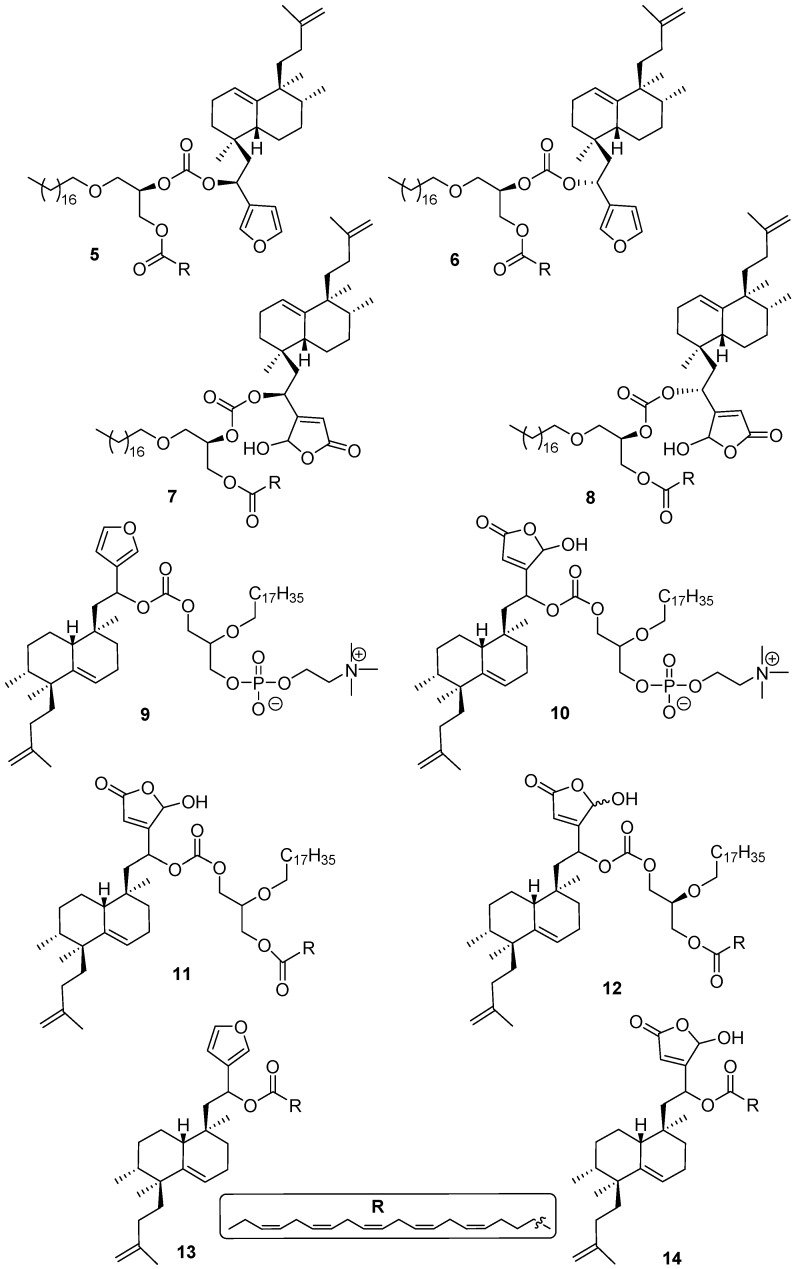
Alkyl glycerol sesterterpenoids bioconjugate compounds **5**–**12** and sesterterpenoid-PUFAs **13**–**14**, synthesized in this work.

The bioconjugates synthesized in this work are displayed in Figure 2, namely: 1-*O*-alkyl-glycerols **5**–**8**, 2-*O*-alkylglycerols **9**–**12** and **13**, **14**. Compounds **5**, **6**, **7** and **8**, synthesized from *R*-solketal, are lipidic ethers (LE) that have in the *sn*2 position of glycerol, a sesterterpenoid joined by a carbonate link, being the glycerol *sn*3 position esterified with an eicosapentaenoic acid (EPA) unit. Compounds **9**, **10**, **11**, **12** show the sesterterpenoid unit in the glycerol *sn*1 position. Bioconjugates **9** and **10** with a phosphocholine unit in *sn*3 of the glycerol and bioconjugates **11** and **12** change the phosphocholine unit for an EPA substituent. Compounds **9**, **10** and **11** were synthesized from racemic solketal and **12** from *S*-solketal. Other bioconjugates, such as **13** and **14** that appear in Figure 2 result from the union of a sesterterpenoid with EPA. The syntheses of all these compounds are described below. We have observed in previous work [46] that the configurational change at C-4 of the sesterterpenoid unit, as in **3** and **4**, does not influence the biological activity, so some bioconjugate compounds have been synthesized and tested without separation of the C-4 epimers. In the same manner, racemic glycerol was used as starting material in the synthesis of several bioconjugates obtained in this work, as the chirality of the glycerol unit did not influence the activity in previous studies on edelfosine derivatives [45,47], so several bioconjugates obtained in this work were prepared using racemic glycerol derivative starting materials.

### 2.1. Synthesis of Bioconjugates ***5**, **6**, **7*** and ***8***

**Scheme 1:**
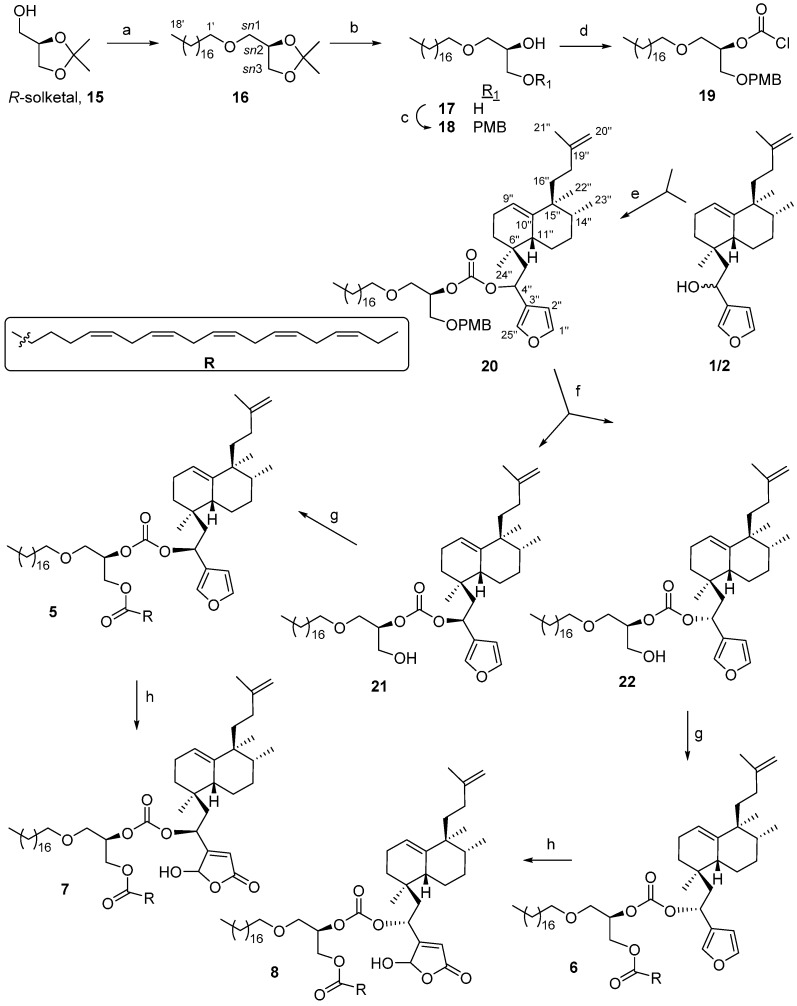
Synthesis of bioconjugates **5**, **6**, **7** and **8**. *Reagents and conditions*: (**a**) Bromooctadecane, NaNH_2_, toluene, 92%; (**b**) *p*-TsOH, MeOH, 40 °C, 93%; (**c**) 1. *n*-Bu_2_SnO, toluene, 2. CsF, PMBCl, DMF, 80%; (**d**) trichloromethylchloroformate, *N*,*N*-dimethylaniline, THF, 83%; (**e**) DMAP, DIPEA, toluene, 60%; (**f**) DDQ, DCM/H_2_O, **21**: 32%, **22**: 65%; (**g**) EPA, EDAC, DMAP, DCM, rt, **5**: 87%, **6**: 82%; (**h**) ^1^O_2_, Rose Bengal, DIPEA, DCM, **7**: 86%, **8**; 90%.

Reaction of *R*-solketal, **15**, (Scheme 1) with bromooctadecane in the presence of NaNH_2_ gives **16**, that by deprotection with *p*-TsOH led to the ether **17**. Regioselective protection of the glycol unit of **17** in the *sn*3 position as the corresponding *p*-methoxybenzyl ether is achieved in good yield, using dibutyltin(IV)oxide and cesium fluoride through a *O*-stannylene acetal intermediate to give **18** [48,49]. This compound reacts with trichloromethyl chloroformate (diphosgene), leading to chlorocarbonate **19**.

The desired carbonate **20**, is obtained by reaction of **19** with the furo-nor-sesterterpenes **1**/**2** in the presence of 4-dimethylaminopyridine (DMAP), and *N*,*N*-diisopropylethylamine (DIPEA). Deprotection of the *p*-methoxybenzyl group of **20** was tried under different conditions (CAN [50], DDQ [51]), achieving the best results when DDQ was used. The obtained hydroxyl derivatives **21** and **22** were separated by column chromatography (CC).

Reaction of **21** and **22** with eicosapentaenoic acid (EPA) [20] (Scheme 1) in the presence of *N*-(3-dimethylaminopropil)-*N*′-ethyl carbodiimide (EDAC) and DMAP, led to **5** and **6**, respectively. These structures were established by studying their NMR spectra. The assignments were corroborated by the [M + Na]^+^ molecular ions observed at 1033.7860 and 1033.7854 for **5** and **6**, respectively, corresponding both to a formula C_66_H_106_O_7_, in agreement with the proposed structures.

Oxidation of **5** and **6** following Faulkner’s methodology [52] (singlet oxygen in the presence of Rose Bengal and DIPEA), gave the γ-hydroxybutenolides **7** and **8** in excellent yield (Scheme 1). The mass spectra of these compounds show molecular ions at 1065.7775 and 1065.7766, which correspond to the formula C_66_H_106_O_9_, thus confirming these structures.

### 2.2. Synthesis of ***9**, **10*** and ***11***

**Scheme 2:**
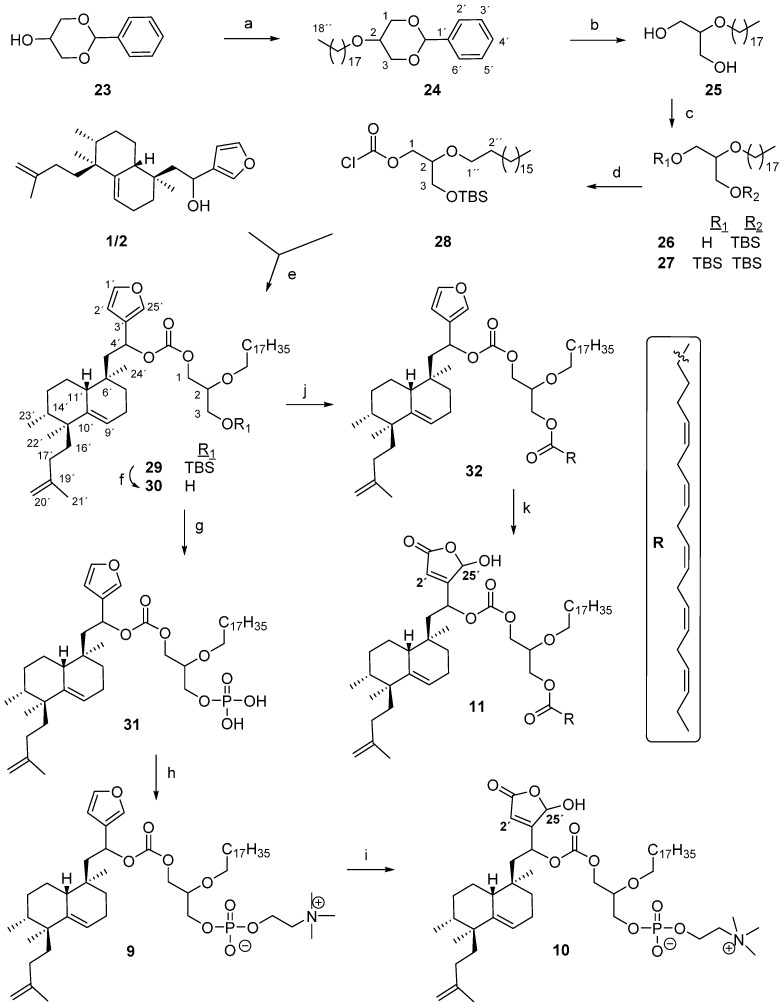
Synthesis of **9**, **10** and **11**. *Reagents and conditions*: (**a**) Bromooctadecane, NaNH_2_, toluene, 98%; (**b**) *p*-TsOH, MeOH, 40 °C, 90%; (**c**) TBDMSCl, imidazole, DMF, rt, **26**: 41%, **27**: 16%, **25**: 42%; (**d**) trichloromethylchloroformate, *N*,*N*-dimethylaniline, THF, 71%; (**e**) DMAP, DIPEA, toluene, 56%; (**f**) TBAF, THF, rt, 89%; (**g**) POCl_3_, pyridine, THF, 0 °C, 97%; (**h**) choline tetraphenylborate, TPS, pyridine, 35%; (**i**) ^1^O_2_, rose bengal, DIPEA, CH_2_Cl_2_, 30%; (**j**) EPA, EDAC, DMAP, CH_2_Cl_2_,rt, 64%; (**k**) ^1^O_2_, Rose Bengal, DIPEA, CH_2_Cl_2_, 53%.

The synthesis of **9**, **10** and **11** was carried out starting from the protected glycerol **23** as shown in Scheme 2. Williamson reaction of the 1,3-*O*-benzylidene glycerol **23** with bromooctadecane and NaNH_2_, led to a nearly quantitative yield of the alkylderivative **24**. Deprotection of **24** with *p*-TsOH gave diol **25** in excellent yield.

Reaction of lipidic ether **25** with *tert*-butyldimethylsilyl chloride (TBDMSCl) and imidazole, rendered a mixture of the starting diol and the monoprotected and diprotected derivatives **26** and **27**, respectively, which were separated by CC. Treatment of **26** with diphosgene in the presence of *N*,*N*-dimethylaniline gave **28** (Scheme 2). Reaction of **28** with the furo-nor-sesterterpenes **1**/**2** in the presence of DMAP and DIPEA lead to **29**. Deprotection of **29** was done with tetrabutylammonium fluoride (TBAF), to obtain the hydroxyderivative **30**, which is the key intermediate in the synthesis of the glycerophosphocholine derivatives **9** and **10** and the bioconjugate **11**. Phosphorylation of **30** was carried out with POCl_3_ in pyridine, affording the phosphatidic acid **31** [18] quantitatively, that was made to react with choline tetraphenylborate [53] and 2,4,6 triisopropylbenzene sulfonyl chloride (TPS) to give **9**. The structure of this compound was established by its NMR spectra. The mass spectrum of **9** shows a [M + Na]^+^ molecular ion at 914.6229 corresponding to C_51_H_90_NO_9_P, corroborating in this manner the structure of the bioconjugate phospholipid. The γ-hydroxy-butenolide **10** was obtained from the furyl derivative **9** by oxidation with singlet oxygen in the presence of Rose Bengal and DIPEA.

Esterification of **30** with EPA, EDAC and DMAP gives the furyl derivative **32** (Scheme 2). Treatment of **32** with singlet oxygen in the presence of Rose Bengal and DIPEA lead to **11**, whose structure was established by its NMR spectra. The mass spectrum of these compounds shows a [M + Na]^+^ molecular ion at 1065.7 corresponding to C_66_H_106_O_9_ in agreement with the proposed structure for compound **11**.

### 2.3. Synthesis of ***12***

**Scheme 3:**
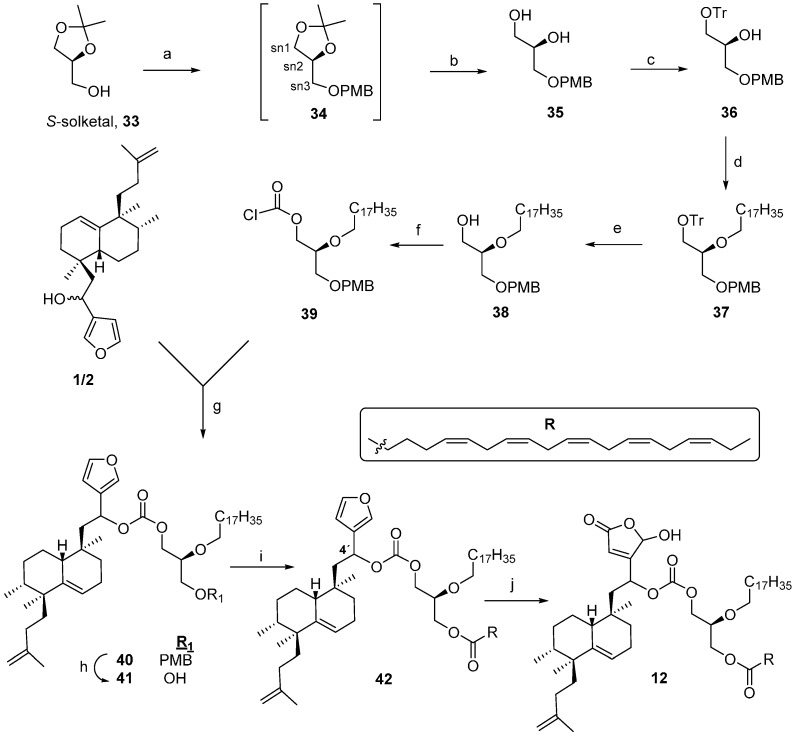
Synthesis of **12**. *Reagents and conditions*: (**a**) PMBCl, NaH, 99%; (**b**) SiO_2_, 90%; (**c**) TrCl, pyridine, 92%; (**d**) bromooctadecane, NaNH_2_, toluene, 97%; (**e**) *p*-TsOH, MeOH, 40 °C, 82%; (**f**) trichloromethylchloroformate, *N*,*N*-dimethylaniline, THF, 82%; (**g**) DMAP, DIPEA, toluene, 58%; (**h**) DDQ, DCM/H_2_O, rt, 71%; (**i**) EPA, EDAC, DMAP, DCM, rt, 63%; (**j**) ^1^O_2_, rose bengal, DIPEA, DCM, 54%.

In order to test the chirality effect, a chiral glycerol was used to obtain compound **12**, the stereoisomer of **11** (Scheme 3). Reaction of *S*-solketal **33** with PMBCl and NaH [54] leads to the *p*-methoxybenzyl derivative **34**, that by chromatography on silica gel is transformed into **35**.

Regioselective protection of **35** is carried out with trityl chloride (TrCl) and pyridine to obtain **36** in an excellent global yield of 83% from *S*-solketal. Alkylation of **36** with bromooctadecane, in the presence of NaNH_2_, gives **37** in good yield. Treatment of **37** with *p*-TsOH [55,56] gives the desired partially deprotected compound **38**. Reaction of **38** with diphosgene in the presence of *N*,*N*-dimethyl-aniline led to chlorocarbonylderivative **39**. Reaction of **39** with the furo-nor-sesterterpenes **1**/**2** mixture in presence of DMAP and DIPEA lead to **40**. Deprotection of **40** was carried out by reaction with DDQ giving **41** (Scheme 3). Esterification of **41** with eicosapentaenoic acid (EPA), in the presence of EDAC and DMAP gives bioconjugate **42**. Oxidation of **42** with singlet oxygen and DIPEA led to compound **12**. The structure of this compound was established by study of its NMR spectra. The mass spectrum of this compound shows a [M + Na]^+^ molecular ion a 1065.7725 corresponding to the molecular formula C_66_H_106_O_9_, corroborating in this manner the structure proposed for compound, **12**.

### 2.4. Synthesis of ***13*** and ***14***

**Scheme 4:**
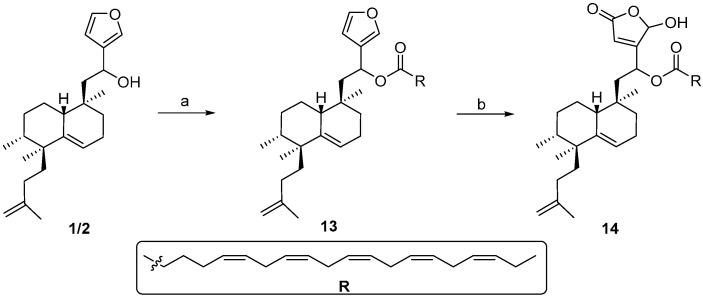
Synthesis of **13** and **14**. *Reagents and conditions*: (**a**) EPA, EDAC, DMAP, DCM, rt, 63%; (**b**) ^1^O_2_, rose bengal, DIPEA, DCM, 54%.

Due to the complexity of the synthesis describe above, the synthesis of simpler bioconjugates, such as **13** and **14** (Scheme 4), was planned in order to obtain more bioconjugate compounds, enabling us to thus do SAR studies. Compound **13** was obtained by direct esterification of **1**/**2** with eicopentaenoic acid (EPA). Reaction of **1**/**2** with eicosapentaenoic acid (EPA) in the presence of EDAC and DMAP leads to compound **13**, that by treatment with singlet oxygen, in the presence of Rose Bengal and DIPEA gives **14**.

## 3. Antitumour Activity of the Bioconjugate Compounds

The *in vitro* antitumour activity for these compounds was determined by measurement of their cytostatic and cytotoxic properties in human tumour cell lines by the XTT assay (Table 1). The cell lines used were HeLa (human epitheloid cervix carcinoma), and MCF7 (human breast carcinoma). Cells were incubated in DMEM (HeLa) or RPMI-1640 (MCF-7) culture medium containing 10% heat-inactivated foetal bovine serum in the absence and in the presence of the indicated compounds at a concentration range of 10^−4^ to 10^−8^ M in a 96-well plate, and following 72 h of incubation at 37 °C in a humidified atmosphere of air/CO_2_ (19:1) the XTT assay was performed as previously described [57].

Measurements were done in triplicate, and the IC_50_ value, defined as the drug concentration required to cause 50% inhibition in the cellular proliferation with respect to the untreated controls, was determined for each compound.

The proliferation inhibition data showed a significant antitumour activity of several compounds as shown in Table 1. When tested compounds **1** and **2** showed less activity against HeLa and MCF7 cells than their γ-hydroxybutenolide counterparts **3** and **4** [46]. This behaviour tells us that the change of a furan fragment for a γ-hydroxybutenolide unit increases the activity, as previously observed by us [46,58,59]. Secondly bioconjugates **5** and **6** are more active than the non-conjugates **1** and **2**, in the same manner bioconjugates **7** and **8** have a better behaviour than **3** and **4** showing than conjugation increases the activity against HeLa and MCF7. These compounds **7** and **8** are more active that edelfosine against HeLa tumour cells and several times better than edelfosine against MCF-7 cells. When changing the sesterterpenoid substitution position on the glycerol unit from secondary as in **7** and **8** to primary as in **9**, **10**, **11** and **12** a light decrease or the same biological activity can be observed in both cell lines. It is remarkable that the activity of these compounds, especially **11** and **12**, duplicates that of the free sesterterpenolides **3** and **4**, so bioconjugation increases the biological activity. When comparing edelfosine against several γ-hydroxybutenolide bioconjugate compounds such as **10**, **11** and **12** on the MCF7 tumour cell line, it can be observed that edelfosine is more active than the phospholipidic ester **10**, while on the contrary, the activity of the γ-hydroxybutenolides **11** and **12** is 6-fold higher than that of edelfosine.

**Table 1:** IC_50_ values of the synthesized compounds against HeLa (human cervix cancer) and MCF7 (human breast cancer) cells. Some natural analogues were included for comparison. Distinct fragments, substitutions or units involved in the structure were included for reference.

Compound	HeLa IC_50_ (μM)	MCF-7 IC_50_ (μM)	Structural Characteristic	Sesterterpene Position	*Sn*3 Substitution
**1/2**	30.2 ± 1.9	32.1 ± 1.1	Furan		
**3**	4.8 ± 0.7	5.1 ± 0.9	α-Hydroxybutenolide		
**4**	5.4 ± 0.6	5.2 ± 1.8	α-Hydroxybutenolide		
**5/6**	13.1 ± 9.5	ND	Furan	*sn*2	EPA
**7**	1.1 ± 0.1	0.6 ± 0.1	α-Hydroxybutenolide	*sn*2	EPA
**8**	1.1 ± 0.1	0.5·± 0.2	α-Hydroxy-butenolide	*sn*2	EPA
**9**	25.0 ± 10	ND	Furan	1	Phosphocholine
**10**	3.3 ± 0.9	6.5 ± 0.8	α-Hydroxybutenolide	1	Phosphocholine
**11**	2.5 ± 0.1	0.6 ± 0.2	α-Hydroxybutenolide	1	EPA
**12**	2.5 ± 0.1	0.5·± 0.1	α-Hydroxybutenolide	*sn*1	EPA
**13**	10.2 ± 3.5	12.3 ± 3.7	Furan	Eicosapentanoylsesterterpene	
**14**	0.3 ± 0.1	0.2 ± 0.1	α-Hydroxybutenolide	Eicosapentanoylsesterterpene	
**Edelfosine**	2.5 ± 0.7	3.1·± 0.9			

Simple bioconjugates **13** and **14** are more biologically active than alkylglycerols **9** and **10** respectively, while in this respect compound **14** is 17 and 26 times more active than γ-hydroxylactones **3** and **4** against HeLa and MCF7 cells, respectively, and more than 40 times more active than eicosapentaenoic acid against HeLa cells [45,46]. Compound **14** is 8 and 15 times more active than edelfosine against HeLa and MCF-7 cells, respectively, making it an interesting starting material for analogue synthesis. In summary the presence of a γ-hydroxybutenolide and simple bioconjugation could be a route to better activity.

## 4. Materials and Methods

### 4.1. General Information

Unless otherwise stated, all chemicals were purchased as the highest purity commercially available and were used without further purification. IR spectra were recorded on an AVATAR 370 FT-IR spectrophotometer (Thermo Nicolet, Salamanca, Spain). ^1^H- and ^13^C-NMR spectra were recorded in CDCl_3_ and referenced to the residual peak of CHCl_3_ at δ 7.26 ppm and δ 77.0 ppm, for ^1^H and ^13^C, respectively, using 200 VX (Varian, Salamanca, Spain) and DRX 400 (Bruker, Salamanca, Spain) instruments. Chemical shifts are reported in δ parts per million and coupling constants (*J*) are given in hertz. MS were recorded using a VG TS 250 spectrometer at 70 eV ionising voltage (Fisons, Salamanca, Spain). Data are presented as *m/z* (% rel. int.). HRMS were recorded on a VG Platform spectrometer using the chemical ionization (ammonia as gas) or fast atom bombardment (FAB) techniques. For some of the samples, a QSTAR XL spectrometer (Evisa, Salamanca, Spain) was employed for electrospray ionization (ESI). Optical rotations were determined on a 241 polarimeter (Perkin-Elmer, Salamanca, Spain) in 1 dm cells. Diethyl ether and THF were distilled from sodium, and dichloromethane was distilled from calcium hydride under argon atmosphere.

### 4.2. Preparation of 1-O-Octadecyl-2,3-isopropyliden-sn-glycerol *(**16**)*

To a solution of (*R*)-(−)-solketal **15** (2.6 g, 19.7 mmol) in toluene (39 mL), NaNH_2_ (768 mg, 19.7 mmol) was added, and the mixture was heated at 111 °C under an argon atmosphere for 1 h. Then it was cooled to rt and a solution of bromooctadecane (6.5 g, 19.7 mmol) in toluene (5 mL) was added, before heating at 111 °C for 3 h. After that time, the reaction mixture was cooled at 0 °C, crushed ice and saturated NH_4_Cl were added and it was extracted with Et_2_O. The organic layer was washed with H_2_O and brine. After drying over anhydrous Na_2_SO_4_, the organic layer was filtered and evaporated. The obtained residue was purified by column chromatography (Hex/EtOAc 9:1) to yield **16** (6.9 g, 92%). ${\left[\alpha \right]}_{D}^{22}$ −8.27 (*c* 1.6, CHCl_3_); IR (film, cm^−1^): 2985, 2924, 2854, 1465, 1369, 1255, 1118, 1057, 849; ^1^H-NMR (400 MHz, CDCl_3_, δ ppm): 4.26 (1H, quin., *J* = 6.0 Hz, H-*sn*2), 4.06 (1H, dd, *J* = 8.2, 6.0 Hz, H_A_-*sn*3), 3.73 (1H, dd, *J* = 8.2, 6.0 Hz, H_B_-*sn*3), 3.51 (1H, dd, *J* = 9.9, 6.0 Hz, H_A_-*sn*1), 3.47 (2H, t, *J* = 6.8 Hz, H-1′), 3.41 (1H, dd, *J* = 9.9, 5.6 Hz, H_B_-*sn*1), 1.56 (2H, m, H-2′), 1.42, 1.36 (3H, s, each, Me_2_C-), 1.25 (30H, m, H-3′-17′), 0.88 (3H, t, *J* = 6.8 Hz, H-18′); ^13^C-NMR (100 MHz, CDCl_3_, δ ppm): 109.3 (Me_2_C-), 74.7 (C-*sn*2), 71.9 (C-1′), 71.8 (C-*sn*1), 66.9 (C-*sn*3), 31.9 (C-16′), 29.4 (C-2′), 29.4 (C-4′-15′), 26.7, 25.4 (Me_2_C-), 26.0 (C-3′), 22.6 (C-17′), 14.0 (C-18′); EIHRMS: calcd. for C_24_H_48_O_3_ [M + H]^+^: 385.3676, found: 385.3680.

### 4.3. Preparation of 1-O-Octadecyl-sn-glycerol *(**17**)*

To a solution of **16** (4.7 g, 12.24 mmol) in MeOH (36 mL), *p*-TsOH (2.3 g, 12.24 mmol) was added and stirred at 35 °C for 8 h. Then H_2_O was added, and the reaction mixture was extracted with Et_2_O and washed with 6% NaHCO_3_ and H_2_O. The organic layer was dried over anhydrous Na_2_SO_4_, filtered and evaporated to give **17** (3.9 g, 11.3 mmol, 93%). ${\left[\alpha \right]}_{D}^{22}$ +0.95 (*c* 0.84, CHCl_3_); IR (film, cm^−1^): 3325, 2918, 2849, 1470 1119, 1063; ^1^H-NMR (400 MHz, CDCl_3_, δ ppm): 3.85 (1H, dddd, *J* = 6.0, 6.0, 4.0, 4.0 Hz, H-*sn*2), 3.71 (1H, dd, *J* = 11.4, 4.0 Hz, H_A_-*sn*3), 3.63 (1H, dd, *J* = 11.4, 6.0 Hz, H_B_-*sn*3), 3.53 (1H, dd, *J* = 9.6, 4.0 Hz, H_A_-*sn*1), 3.49 (1H, dd, *J* = 9.6, 6.0 Hz, H_B_-*sn*1), 3.47 (1H, ddd, *J* = 9.3, 6.7, 6.7 Hz, H_A_-1′), 3.44 (1H, ddd, *J* = 9.3, 6.7, 6.7 Hz, H_B_-1′), 1.56 (2H, m, H-2′), 1.25 (30H, m, H-3′-17′), 0.87 (3H, t, *J* = 6.8 Hz, H-18′); ^13^C-NMR (100 MHz, CDCl_3_, δ ppm): 72.4 (C-*sn*1), 71.8 (C-1′), 70.4 (C-*sn*2), 64.2 (C-*sn*3), 31.8 (C-16′), 29.5 (C-2′), 29.5 (C-4′-15′), 26.0 (C-3′), 22.6 (C-17′), 14.0 (C-18′); EIHRMS: calcd. for C_21_H_44_O_3_ [M + Na]^+^: 367.3183, found: 367.3194.

### 4.4. Preparation of 1-O-Octadecyl-3-O-p-methoxybenzyl-sn-glycerol *(**18**)*

To a solution of **17** (200 mg, 0.58 mmol) in toluene (3.4 mL), dibutyl tin (IV) oxide (144 mg, 0.58 mmol) was added and it was heated up to reflux for 2 h in a Dean-Stark apparatus. After this time, the solvent was evaporated to give a white solid, and CsF (167 mg, 1.1 mmol) was added to this solid. The solid mixture was dried for 1 h 30 min under high vacuum. It was then diluted in DMF (3.4 mL) and PMBCl (258 mg, 1.65 mmol) added and stirred overnight under an argon atmosphere. Then H_2_O (1 mL) and EtOAc (3 mL) were added, the reaction mixture was stirred vigorously for 15 min and filtered through a pad of silica gel to remove the dibutyl tin oxide. The filtrate was washed with H_2_O and brine. Removal of the solvents gave a residue that was purified by column chromatography (Hex/EtOAc 96:4) to obtain **18** (215 mg, 80%). ${\left[\alpha \right]}_{D}^{22}$ +1.2 (*c* 0.11, CHCl_3_); IR (film, cm^−1^): 3485, 3404, 2916, 2846, 1470, 1031; ^1^H-NMR (400 MHz, CDCl_3_, δ ppm): 7.27 (2H, d, *J* = 7.4 Hz, H-2′′′, H-6′′′), 6.89 (2H, d, *J* = 7.4 Hz, H-3′′′, H-5′′ ), 4.50 (2H, s, –OCH_2_Ar), 3.98 (1H, quin, *J* = 4.8 Hz, H-*sn*2), 3.82 (3H, s, –OMe), 3.65–3.55 (6H, m, H-*sn*1, 1′, *sn*3), 1.56 (2H, m, H-2′), 1.28 (30H, m, H-3′-17′), 0.89 (3H, t, *J* = 6.2 Hz, H-18′); ^13^C-NMR (100 MHz, CDCl_3_, δ ppm): 159.2 (C-4′′′), 130.1 (C-1′′′), 129.3 (C-2′′′), 129.3 (C-6′′′), 113.8 (C-3′′′), 113.8 (C-5′′′), 73.1 (–OCH_2_Ar), 71.7 (C-1′), 71.6 (C-*sn*1), 71.0 (C-*sn*3), 69.5 (C-*sn*2), 55.2 (C–OMe), 31.9 (C-16′), 29.5 (C-2′), 29.5 (C-4′-15′), 26.1 (C-3′), 22.7 (C-17′), 14.1 (C-18′); EIHRMS: calcd. for C_29_H_52_O_4_ [M + Na]^+^: 487.3758, found: 487.3773.

### 4.5. Preparation of 1-O-Octadecyl-2-chlorocarbonyl-3-O-p-methoxybenzyl-sn-glycerol *(**19**)*

To an ice cooled solution of **18** (377 mg, 0.81 mmol) in THF (1.6 mL), trichloromethyl chloroformate (diphosgene, 160 mg, 0.81 mmol) and *N*,*N*-dimethylaniline (98 mg, 0.81 mmol) were added. The mixture was stirred at 0 °C for 10 min and then at rt overnight. Then Et_2_O was added and the white precipitate formed was filtered. The solution washed with 0.2 M HCl, 0.2 M NaOH and H_2_O, then dried over anhydrous Na_2_SO_4_ and evaporated to give **19** (353 mg, 0.67 mmol, 83%). IR (film, cm^−1^): 2924, 2852, 1780, 1166; ^1^H-NMR (200 MHz, CDCl_3_, δ ppm): 7.24 (1H, d, *J* = 7.0 Hz, each, H-2′′′, H-6′′′), 6.89 (1H, d, *J* = 7.4 Hz, each, H-3′′′, H-5′′′), 5.16 (1H, quin, *J* = 4.8 Hz, H-*sn*2), 4.49 (2H, s, –OCH_2_Ar), 3.81 (3H, s, –OMe), 3.64–3.59 (4H, m, H-*sn*1, *sn*3), 3.44–3.38 (2H, m, H-1′), 1.54 (2H, m, H-2′), 1.25 (30H, m, H-3′-17′), 0.89 (3H, t, *J* = 6.2 Hz, H-18′); ^13^C-NMR (50 MHz, CDCl_3_, δ ppm): 159.2 (C-4′′′), 154.9 (–O–CO–Cl), 129.8 (C-1′′′), 129.5 (C-2′′′, 6′′′), 114.1 (C-3′′′, 5′′′), 80.5 (C-*sn*2), 73.3 (–OCH_2_Ar), 72.1 (C-1′), 68.9 (C-*sn*1), 67.9 (C-*sn*3), 55.5 (C–OMe), 32.2 (C-16′), 29.8 (C-2′), 29.8 (C-4′-15′), 26.2 (C-3′), 22.9 (C-17′), 14.3 (C-18′); EIHRMS: in MeOH, calcd. for methyl ester, C_31_H_54_O_6_ [M + Na]^+^: 545.3813, found: 545.3808.

### 4.6. Preparation of 1-O-Octadecyl-2-O-[1,25-epoxy-18-nor-ent-isodysidiola-1,3(25),9,19-tetraen-4R/S-yloxycarbonyl]-3-p-methoxybenzyl-sn-glycerol *(**20**)*

To a solution of **1**/**2** (153 mg, 0.43 mmol), *N*,*N*-diisopropylethylamine (DIPEA, 71 mg, 0.55 mmol), 4-(dimethylamino) pyridine (DMAP, 26 mg, 0.21 mmol) in toluene (2.1 mL), a solution of **19** (172 mg, 0.33 mmol) in toluene (1.65 mL) was added dropwise at 0 °C . The reaction mixture was stirred at 0 °C under an argon atmosphere for 15 min and then at rt overnight. After this time the solvent was removed and the residue was purified by column chromatography (Hex/EtOAc 99:1) to obtain **20** (167 mg, 60%). IR (film, cm^−1^): 2924, 2853, 1744, 1514, 1464, 1258, 1115; ^1^H-NMR (400 MHz, CDCl_3_, δ ppm): 7.42/7.41 (1H, s, H-25′′), 7.34/7.33 (1H, s, H-1′′), 7.24/7.17 (2H, d, *J* = 8.8 Hz, H-2′′′, H-6′′′), 6.86/6.83 (2H, d, *J* = 8.8 Hz, H-3′′′, H-5′′′), 6.39 (1H, s, H-2′′), 5.81/5.79 (1H, dd, *J* = 5.6, 3.2 Hz, H-4′′), 5.35/5.33 (1H, t, *J* = 3.2 Hz, H-9′′), 4.98 (1H, m, H-*sn*2), 4.65–4.62 (2H, m, H-20′′), 4.49/4.45 (1H, d, *J* = 11.6 Hz, –OCH_2_Ar), 4.44/4.39 (1H, d, *J* = 11.6 Hz, –OCH_2_Ar), 3.80 (3H, s, –OMe), 3.64–3.50 (2H, m, H-*sn*1), 3.64–3.50 (2H, m, H-*sn*3), 3.47–3.31 (2H, m, H-1′), 2.20–1.40 (16H, m, H-5′′, 7′′, 8′′, 11′′, 12′′, 13′′, 14′′, 16′′, 17′′), 1.70/1.67 (3H, s, Me-21′′), 1.56 (2H, m, H-2′), 1.26 (30H, m, H-3′-17′), 0.91 (3H, s, Me-22′′), 0.89 (3H, t, *J* = 6.2 Hz, Me-18′), 0.88 (3H, s, Me-24′′), 0.81/0.80 (3H, d, *J* = 7.0 Hz, Me-23′′); ^13^C-NMR (100 MHz, CDCl_3_, δ ppm): 159.2 (C-4′′′), 154.2 (–O–CO–O–), 147.0/146.9 (C-19′′), 143.1 (C-1′′), 141.4/141.0 (C-10′′), 140.0/139.9 (C-25′′), 130.0 (C-1′′′), 129.2 (C-2′′′, 6′′′), 126.2 (C-3′′), 120.0/119.7 (C-9′′), 113.7 (C-3′′′, 5′′′), 109.1/109.0 (C-20′′), 108.8/108.7 (C-2′′), 75.5/75.4 (C-*sn*2), 72.9 (–OCH_2_Ar), 71.7/71.6 (C-1′), 70.0 (C-4′′), 69.2/69.1 (C-*sn*1), 68.5/68.3 (C-*sn*3), 55.2 (–OMe), 44.3 (C-5′′), 42.9 (C-15′′), 42.6 (C-11′′), 38.9/38.7 (C-14′′), 37.4/37.2 (C-16′′), 34.1/34.0 (C-6′′), 32.4/32.3 (C-17′′), 31.9 (C-16′), 29.6–28.9 (C-2′), 29.6–28.9 (C-4′-15′), 29.6–28.9 (C-7′′, 13′′), 26.0/25.9 (C-3′), 23.2 (C-8′′, 12′′), 22.8 (C-24′′), 22.6 (C-17′), 22.3 (C-21′′), 22.2 (C-22′′), 15.6 (C-23′′), 14.0 (C-18′); EIHRMS: calcd. for C_54_H_86_O_7_ [M + Na]^+^: 869.6266, found: 869.6233.

### 4.7. Reaction of Compound ***20*** with DDQ: Preparation of 1-O-Octadecyl-2-O-[1,25-epoxy-18-nor-ent-isodysidiola-1,3(25),9,19-tetraen-4S-yloxycarbonyl]-sn-glycerol *(**21**)* and 1-O-Octadecyl-2-O-[1,25-epoxy-18-nor-ent-isodysidiola-1,3(25),9,19-tetraen-4R-yloxycarbonyl]-sn-glycerol *(**22**)*

To a solution of **20** (170 mg, 0.2 mmol) in CH_2_Cl_2_/H_2_O 18:1 (2.2 mL), DDQ (54 mg, 0.24 mmol) was added. The reaction mixture was stirred at rt under an argon atmosphere for 1 h 15 min, quenched with 6% NaHCO_3_ and extracted with CH_2_Cl_2_. The organic layer was washed with 6% NaHCO_3_ and brine and dried over anhydrous Na_2_SO_4_. Removal of the solvent gave the crude product which was purified by column chromatography on silica gel to obtain **21** (46 mg, 32%, Hex/EtOAc 97:3 as eluent) and **22** (94 mg, 65%, Hex/EtOAc 95:5 as eluent).

*Compound* **21**: ${\left[\alpha \right]}_{D}^{22}$ +42.2 (*c* 0.46, CHCl_3_); IR (film, cm^−1^): 3464, 2924, 2853, 1744, 1260; ^1^H-NMR (400 MHz, CDCl_3_, δ ppm): 7.43 (1H, s, H-25′′), 7.35 (1H, s, H-1′′), 6.40 (1H, s, H-2′′), 5.79 (1H, dd, *J* = 8.5, 3.6 Hz, H-4′′), 5.33 (1H, t, *J* = 4.8 Hz, H-9′′), 4.81 (1H, quin, *J* = 5.0 Hz, H-*sn*2), 4.65 and 4.64 (1H, s, each, H-20′′), 3.84 (1H, dd, *J* = 12.0, 5.0 Hz, H_A_-*sn*3), 3.79 (1H, dd, *J* = 12.0, 5.0 Hz, H_B_-*sn*3), 3.60 (1H, dd, *J* = 10.8, 5.0 Hz, H_A_-*sn*1), 3.56 (1H, dd, *J* = 10.8, 5.0 Hz, H_B_-*sn*1), 3.39 (2H, m, H-1′), 2.05–1.4 (16H, m, H-5′′, 7′′, 8′′, 11′′, 12′′, 13′′ 14′′, 16′′, 17′′), 1.70 (3H, s, Me-21′′), 1.56 (2H, m, H-2′), 1.25 (30H, m, H-3′-17′), 0.90 (3H, s, Me-22′′), 0.89 (3H, s, Me-24′′), 0.88 (3H, t, *J* = 7.2 Hz, Me-18′), 0.80 (3H, d, *J* = 7.0 Hz, Me-23′′); ^13^C-NMR (100 MHz, CDCl_3_, δ ppm): 154.2 (–O–CO–O–), 147.1 (C-19′′), 143.2 (C-1′′), 141.4 (C-10′′), 140.0 (C-25′′), 126.0 (C-3′′), 119.7 (C-9′′), 109.1 (C-20′′), 108.6 (C-2′′), 76.6 (C-*sn*2), 71.9 (C-1′), 70.4 (C-4′′), 69.5 (C-*sn*1), 62.6 (C-*sn*3), 44.2 (C-5′′), 42.9 (C-15′′), 42.4 (C-11′′), 38.7 (C-14′′), 37.4 (C-16′′), 34.0 (C-6′′), 32.4 (C-17′′), 31.9 (C-16′), 31.0 (C-7′′), 29.6–29.3 (C-2′), 29.6–29.3 (C-4′-15′), 28.8 (C-13′′), 25.9 (C-3′), 22.8 (C-12′′), 22.7 (C-24′′), 22.6 (C-8′′), 22.6 (C-17′), 22.5 (C-21′′), 22.3 (C-22′′), 15.6 (C-23′′), 14.1 (C-18′); EIHRMS: calcd. for C_46_H_78_O_6_ [M + Na]^+^: 749.5691, found: 749.5706.

*Compound* **22**: ${\left[\alpha \right]}_{D}^{22}$ +3.5 (*c* 0.40, CHCl_3_); IR (film, cm^−1^): 3477, 2924, 2853, 1742, 1261; ^1^H-NMR (400 MHz, CDCl_3_, δ ppm): 7.43 (1H, s, H-25′′), 7.35 (1H, s, H-1′′), 6.40 (1H, s, H-2′′), 5.80 (1H, dd, *J* = 8.4, 3.4 Hz, H-4′′), 5.36 (1H, t, *J* = 3.4 Hz, H-9′′), 4.83 (1H, quin, *J* = 5.0 Hz, H-*sn*2), 4.65, 4.61 (1H, s, each, H-20′′), 3.79 (2H, m, H-*sn*3), 3.62 (1H, dd, *J* = 10.6, 5.0 Hz, H_A_-*sn*1), 3.60 (1H, dd, *J* = 10.6, 5.0 Hz, H_B_-*sn*1), 3.44 (2H, m, H-1′), 2.15–1.4 (16H, m, H-5′′, 7′′, 8′′, 11′′, 12′′, 13′′ 14′′, 16′′, 17′′), 1.67 (3H, s, Me-21′′), 1.56 (2H, m, H-2′), 1.25 (30H, m, H-3′-17′), 0.91 (3H, s, Me-22′′), 0.88 (3H, s, Me-24′′), 0.86 (3H, t, *J* = 6.2 Hz, Me-18′), 0.81 (3H, d, *J* = 7.0 Hz, Me-23′′); ^13^C-NMR (100 MHz, CDCl_3_, δ ppm): 154.2 (–O–CO–O–), 147.0 (C-19′′), 143.2 (C-1′′), 141.0 (C-10′′), 140.2 (C-25′′), 125.9 (C-3′′), 120.0 (C-9′′), 109.0 (C-20′′), 108.7 (C-2′′), 76.9 (C-*sn*2), 71.9 (C-1′), 70.3 (C-4′′), 69.5 (C-*sn*1), 62.6 (C-*sn*3), 44.2 (C-5′′), 42.9 (C-15′′), 42.3 (C-11′′), 38.8 (C-14′′), 37.2 (C-16′′), 34.1 (C-6′′), 32.3 (C-7′′), 32.3 (C-17′′), 31.9 (C-16′), 29.6–29.3 (C-2′), 29.6–29.3 (C-4′-15′), 28.9 (C-13′′), 26.0 (C-3′), 23.2 (C-12′′), 22.8 (C-24′′), 22.6 (C-8′′), 22.6 (C-17′), 22.2 (C-21′′), 22.2 (C-22′′), 15.6 (C-23′′), 14.1 (C-18′); EIHRMS: calcd. for C_46_H_78_O_6_ [M + Na]^+^: 749.5691, found: 749.5666.

### 4.8. Preparaion of 1-O-Octadecyl-2-O-[1,25-epoxy-18-nor-ent-isodysidiola-1,3(25),9,19-tetraen-4S-yloxycarbonyl]-3-eicosapentaenoyl-sn-glycerol *(**5**)*

To a solution of **21** (10 mg, 0.01 mmol), DMAP (3 mg, 0.02 mmol) and EDAC (3.5 mg, 0.02 mmol) in dry CH_2_Cl_2_ (0.14 mL), EPA (4.2 mg, 0.01 mmol) was added under an argon atmosphere. After stirring at rt for 12 h, the reaction mixture was passed through a short silica gel column (CH_2_Cl_2_/EtOAc 9:1 as eluent). Then the solvent was removed and the crude oil was purified by column chromatography (Hex/EtOAc 98:2) providing **5** (12 mg, 87%). ${\left[\alpha \right]}_{D}^{22}$ +7.5 (*c* 0.20, CHCl_3_); IR (film, cm^−1^): 2957, 2926, 2855, 1745, 1462, 1261; ^1^H-NMR (400 MHz, CDCl_3_, δ ppm): 7.42 (1H, s, H-25′′), 7.35 (1H, s, H-1′′), 6.39 (1H, s, H-2′′), 5.79 (1H, dd, *J* = 8.7, 3.1 Hz, H-4′′), 5.41–5.32 (10H, m, =CH), 5.32 (1H, m, H-9′′), 5.01 (1H, m, H-*sn*2), 4.65, 4.63 (1H, s, each, H-20′′), 4.36 (1H, dd, *J* = 12.0, 3.4 Hz, H_A_-*sn*3), 4.15 (1H, dd, *J* = 12.0, 6.7 Hz, H_B_-*sn*3), 3.51 (2H, d, *J* = 5.4 Hz, H-*sn*1), 3.40–3.34 (2H, m, H-1′), 2.85–2.80 (8H, m, =CCH_2_C=), 2.31 (2H, t, *J* = 7.3 Hz, H-2′′′), 2.09–2.04 (4H, m, H-4′′′, 19′′′), 2.05–1.40 (16H, m, H-5′′, 7′′, 8′′, 11′′, 12′′, 13′′, 14′′, 16′′, 17′′), 1.76–1.72 (2H, m, H-3′′′), 1.70 (3H, s, Me-21′′), 1.56 (2H, m, H-2′), 1.25 (30H, m, H-3′-17′), 0.97 (3H, t, *J* = 7.5 Hz, H-20′′′), 0.90 (3H, s, Me-22′′), 0.89 (3H, t, *J* = 6.8 Hz, Me-18′), 0.88 (3H, s, Me-24′′), 0.80 (3H, d, *J* = 6.8 Hz, Me-23′′); ^13^C-NMR (100 MHz, CDCl_3_, δ ppm): 173.4 (C-1′′′), 154.2 (–O–CO–O–), 147.3 (C-19′′), 143.5 (C-1′′), 141.7 (C-10′′), 140.0 (C-25′′), 132.3 (C-18′′′), 129.1–127.2 (=CH) × 9, 126.2 (C-3′′), 119.9 (C-9′′), 109.4 (C-20′′), 108.9 (C-2′′), 74.3 (C-*sn*2), 72.1 (C-1′), 70.5 (C-4′′), 68.9 (C-*sn*1), 63.1 (C-*sn*3), 44.4 (C-5′′), 43.1 (C-15′′), 42.9 (C-11′′), 39.1 (C-14′′), 37.6 (C-16′′), 34.3 (C-6′′), 33.7 (C-2′′′), 32.7 (C-17′′), 32.1 (C-16′), 31.1 (C-7′′), 29.9–29.3 (C-2′), 29.9–29.3 (C-4′-15′), 29.1 (C-13′′), 28.7–20.8 (3′′′, 4′′′, 7’’’, 10’’’, 13’’’, 16’’’, 19′′′), 26.0 (C-3′), 24.0 (C-24′′), 22.9 (C-12′′), 22.9 (C-21′′), 22.6 (C-8′′), 22.6 (C-17′), 22.5 (C-22′′), 15.9 (C-23′′), 14.5 (C-20′′′), 14.3 (C-18′); EIHRMS: calcd. for C_66_H_106_O_7_ [M + Na]^+^: 1033.7831, found: 1033.7860.

### 4.9. Preparation of 1-O-Octadecyl-2-O-[1,25-epoxy-18-nor-ent-isodysidiola-1,3(25),9,19-tetraen-4R-yloxycarbonyl]-3-eicosapentaenoyl-sn-glycerol *(**6**)*

To a solution of **22** (12.5 mg, 0.02 mmol), DMAP (3 mg, 0.02 mmol) and EDAC (4 mg, 0.02 mmol) in dry CH_2_Cl_2_ (0.2 mL), EPA (5.2 mg, 0.02 mmol) was added under an argon atmosphere. After stirring at rt for 12 h, the reaction mixture was passed through a short silica gel column (CH_2_Cl_2_/EtOAc 9:1 as eluent). Then the solvent was removed and the crude was purified by column chromatography (Hex/EtOAc 99:1) providing **6** (14 mg, 82%). ${\left[\alpha \right]}_{D}^{22}$ +2.4 (*c* 0.33, CHCl_3_); IR (film, cm^−1^): 2959, 2924, 2855, 1744, 1263; ^1^H-NMR (400 MHz, CDCl_3_, δ ppm): 7.42 (1H, s, H-25′′), 7.33 (1H, s, H-1′′), 6.39 (1H, s, H-2′′), 5.79 (1H, dd, *J* = 8.5, 3.0 Hz, H-4′′), 5.40–5.34 (10H, m, =CH), 5.34 (1H, m, H-9′′), 5.04–5.00 (1H, m, H-*sn*2), 4.65 and 4.61 (1H, s, each, H-20′′), 4.30 (1H, dd, *J* = 12.1, 3.6 Hz, H_A_-*sn*3), 4.15 (1H, dd, *J* = 12.1, 7.0 Hz, H_B_-*sn*3), 3.56 (1H, dd, *J* = 12.2, 5.4 Hz, H_A_-*sn*1), 3.52 (1H, dd, *J* = 14.2, 5.4 Hz, H_B_-*sn*1), 3.40 (2H, m, H-1′), 2.85–2.78 (8H, m, =CCH_2_C=), 2.21 (2H, t, *J* = 7.4 Hz, H-2′′′), 2.13–2.04 (4H, m, H-4′′′, 19′′′), 2.05–1.40 (16H, m, H-5′′, 7′′, 8′′, 11′′, 12′′, 13′′, 14′′, 16′′, 17′′), 1.76–1.72 (2H, m, H-3′′′), 1.68 (3H, s, Me-21′′), 1.56 (2H, m, H-2′), 1.25 (30H, m, H-3′-17′), 0.97 (3H, t, *J* = 7.5 Hz, H-20′′′), 0.90 (3H, t, *J* = 6.8 Hz, Me-18′), 0.88 (3H, s, Me-22′′), 0.86 (3H, s, Me-24′′), 0.81 (3H, d, *J* = 7.0 Hz, Me-23′′); ^13^C-NMR (100 MHz, CDCl_3_, δ ppm): 173.1 (C-1′′′), 154.2 (–O–CO–O–), 146.9 (C-19′′), 143.1 (C-1′′), 141.0 (C-10′′), 140.1 (C-25′′), 132.0 (C-18′′′), 128.9–127.0 (=CH) × 9, 126.1 (C-3′′), 120.0 (C-9′′), 109.0 (C-20′′), 108.7 (C-2′′), 74.0 (C-*sn*2), 71.8 (C-1′), 70.3 (C-4′′), 68.8 (C-*sn*1), 62.7 (C-*sn*3), 44.3 (C-5′′), 42.9 (C-15′′), 42.3 (C-11′′), 38.8 (C-14′′), 37.6 (C-16′′), 34.1 (C-6′′), 33.3 (C-2′′′), 32.2 (C-17′′), 31.9 (C-16′), 31.2 (C-7′′), 29.6–29.3 (C-2′), 29.6–29.3 (C-4′-15′), 28.8 (C-13′′), 28.4–22.6 (3′′′, 4′′′, 7′′′, 10′′′, 13′′′, 16′′′, 19′′′), 26.0 (C-3′), 23.7 (C-24′′), 23.2 (C-12′′), 22.8 (C-21′′), 22.6 (C-8′′), 22.6 (C-17′), 22.2 (C-22′′), 15.6 (C-23′′), 14.2 (C-20′′′), 14.0 (C-18′); EIHRMS: calcd. for C_66_H_106_O_7_ [M + Na]^+^: 1033.7831, found: 1033.7854.

### 4.10. Preparation of 1-O-Octadecyl-2-O-[25-hydroxy-18-nor-ent-isodysidiola-2,9,19-trien-1,25-olide-4S-yloxycarbonyl]-3-eicosapentaenoyl-sn-glycerol *(**7**)*

Rose Bengal (1 mg) was added to a solution of **5** (6.4 mg, 6.3 × 10^−3^ mmol) and DIPEA (11 μL, 0.06 mmol) in dry CH_2_Cl_2_ (2 mL) at rt. Anhydrous oxygen was bubbled in for 2 min and after that, the solution was placed under an oxygen atmosphere at −78 °C and irradiated with a 200 W lamp. After 4 h irradiation was stopped, the pink solution was allowed to warm to rt, and saturated aqueous oxalic acid solution (1 mL) added. After a few minutes of vigorous stirring, the mixture was diluted with H_2_O and extracted with Et_2_O. The combined organic extracts were washed with H_2_O and dried over anhydrous Na_2_SO_4_. After filtration, the solvent was evaporated to give a residue that was purified by silica gel column chromatography to yield **7** (6 mg, 86%). ${\left[\alpha \right]}_{D}^{22}$ +2.6 (*c* 0.2, CHCl_3_); IR (film, cm^−1^): 3427, 2924, 1747, 1259; ^1^H-NMR (400 MHz, CDCl_3_, δ ppm): 6.19/5.97 (1H, m, H-25′′), 6.01/6.00 (1H, m, H-2′′), 5.60/5.48 (1H, m, H-4′′), 5.45–5.33 (10H, m, =CH), 5.45–5.33 (1H, m, H-9′′), 5.03/4.95 (1H, m, H-*sn*2), 4.68–4.62 (2H, m, H-20′′), 4.35/4.18 (2H, m, H-*sn*3), 3.56 (2H, m, H-*sn*1), 3.43 (2H, m, H-1′), 2.82 (8H, m, =CCH_2_C=), 2.30 (2H, m, H-2′′′), 2.09 (4H, m, H-4′′′, 19′′′), 2.00–1.53 (18H, m, H-5′′, 7′′, 8′′, 11′′, 12′′, 13′′, 14′′, 16′′, 17′′, 3′′′), 1.69 (3H, s, Me-21′′), 1.54 (2H, m, H-2′), 1.25 (30H, m, H-3′-17′), 0.97 (3H, t, *J* = 7.6 Hz, H-20′′′), 0.91 (3H, s, Me-22′′), 0.88 (3H, t, *J* = 6.8 Hz, Me-18′), 0.88 (3H, s, Me-24′′), 0.81 (3H, d, *J* = 6.8 Hz, Me-23′′); ^13^C-NMR (50 MHz, CDCl_3_, δ ppm): 173.7 (C-1′′′), 169.4 (C-1′′), 168.2 (C-3′′), 154.4 (–O–CO–O–), 147.5 (C-19′′), 141.8 (C-10′′), 132.3 (C-18′′′), 129.2–127.2 (=CH) × 9, 120.0 (C-9′′), 117.9 (C-2′′), 109.4 (C-20′′), 97.6 (C-25′′), 74.3 (C-*sn*2), 72.1 (C-1′), 71.2 (C-4′′), 68.9 (C-*sn*1), 63.0 (C-*sn*3), 43.1 (C-5′′), 42.9 (C-15′′), 42.9 (C-11′′), 38.9 (C-14′′), 37.6 (C-16′′), 34.4 (C-6′′), 33.7 (C-2′′′), 32.7 (C-17′′), 32.1 (C-16′), 31.1 (C-7′′), 29.9–29.3 (C-2′), 29.9–29.3 (C-4′-15′), 29.1 (C-13′′), 28.6–20.7 (3′′′, 4′′′, 7′′′, 10′′′, 13′′′, 16′′′, 19′′′), 25.9 (C-3′), 24.0 (C-24′′), 22.9 (C-12′′), 22.9 (C-21′′), 22.6 (C-8′′), 22.6 (C-17′), 22.5 (C-22′′), 15.9 (C-23′′), 14.5 (C-20′′′), 14.3 (C-18′); EIHRMS: calcd. for C_66_H_106_O_9_ [M + Na]^+^: 1065.7729, found: 1065.7775.

### 4.11. Preparation of 1-O-Octadecyl-2-O-[25-hydroxy-18-nor-ent-isodysidiola-2,9,19-trien-1,25-olide-4R-yloxycarbonyl]-3-eicosapentaenoyl-sn-glycerol *(**8**)*

Rose Bengal (1 mg) was added to a solution of **6** (7.6 mg, 7.5 × 10^−3^ mmol) and DIPEA (13 μL, 0.075 mmol) in dry CH_2_Cl_2_ (2 mL) at rt. Anhydrous oxygen was bubbled in for 2 min, then the solution was placed under an oxygen atmosphere at −78 °C and irradiated with a 200 W lamp. After 4 h irradiation was stopped, the pink solution allowed to warm to rt, and saturated aqueous oxalic acid solution (1 mL) added. After a few minutes of vigorous stirring, the mixture was diluted with H_2_O and extracted with Et_2_O. The combined organic extracts were washed with H_2_O and dried over anhydrous Na_2_SO_4_. After filtration, the solvent was evaporated to give a residue which was purified by silica gel column chromatography to yield **8** (7 mg, 90%). ${\left[\alpha \right]}_{D}^{22}$ −3.0 (*c* 0.3, CHCl_3_); ); IR (film, cm^−1^): 3427, 2924, 1747, 1259; ^1^H-NMR (400 MHz, CDCl_3_, δ ppm): 6.13/5.96 (1H, m, H-25′′), 6.06/6.05 (1H, s, H-2′′), 5.69/5.46 (1H, m, H-4′′), 5.40–5.34 (10H, m, =CH), 5.40–5.34 (1H, m, H-9′′), 5.01/4.94 (1H, m, H-*sn*2), 4.67–4.63 (2H, m, H-20′′), 4.13 (1H, dd, *J* = 6.4, 12.4 Hz, H_A_-*sn*3), 4.01 (1H, dd, *J* = 4.0, 12.4 Hz, H_B_-*sn*3), 3.57 (2H, d, *J* = 5.2 Hz, H-*sn*1), 3.47–3.39 (2H, m, H-1′), 2.86–2.80 (8H, m, =CCH_2_C=), 2.34–2.30 (2H, m, H-2′′′), 2.13–2.04 (4H, m, H-4′′′, 19′′′), 2.00–1.52 (18H, m, H-5′′, 7′′, 8′′, 11′′, 12′′, 13′′, 14′′, 16′′, 17′′, 3′′′), 1.69 (3H, s, Me-21′′), 1.54 (2H, m, H-2′), 1.25 (30H, m, H-3′-17′), 0.97 (3H, t, *J* = 7.6 Hz, H-20′′′), 0.91 (3H, s, Me-22′′), 0.88 (3H, t, *J* = 6.8 Hz, Me-18′), 0.88 (3H, s, Me-24′′), 0.81 (3H, d, *J* = 6.8 Hz, Me-23′′); ^13^C-NMR (100 MHz, CDCl_3_, δ ppm): 173.4 (C-1′′′), 169.3 (C-1′′), 168.1 (C-3′′), 154.4 (–O–CO–O–), 147.1 (C-19′′), 141.1 (C-10′′), 132.0 (C-18′′′), 128.8–127.0 (=CH) × 9, 120.0 (C-9′′), 118.0 (C-2′′), 109.0 (C-20′′), 97.5 (C-25′′), 74.0 (C-*sn*2), 71.8 (C-1′), 71.1 (C-4′′), 68.8 (C-*sn*1), 62.6 (C-*sn*3), 42.9 (C-5′′), 42.9 (C-15′′), 42.3 (C-11′′), 38.8 (C-14′′), 37.6 (C-16′′), 34.1 (C-6′′), 33.3 (C-2′′′), 32.2 (C-17′′), 31.9 (C-16′), 31.2 (C-7′′), 29.7–29.3 (C-2′), 29.7–29.3 (C-4′-15′), 28.8 (C-13′′), 28.3–22.6 (3′′′, 4′′′, 7′′′, 10′′′, 13′′′, 16′′′, 19′′′), 26.0 (C-3′), 23.7 (C-24′′), 23.2 (C-12′′), 22.8 (C-21′′), 22.6 (C-8′′), 22.6 (C-17′), 22.2 (C-22′′), 15.7 (C-23′′), 14.2 (C-20′′′), 14.0 (C-18′); EIHRMS: calcd. for C_66_H_106_O_9_ [M + Na]^+^: 1065.7729, found: 1065.7766.

### 4.12. Preparation of 1,3-Benzyliden-2-O-octadecylglycerol *(**24**)*

To a solution of 1,3-*O*-benzylidene glycerol **23** (3.7 g, 20.5 mmol) in toluene (21 mL), NaNH_2_ (800 mg, 20.5 mmol) was added and heated at 111 °C under an argon atmosphere for 1 h. The mixture was cooled to rt and a solution of bromooctadecane (6.8 g, 20.5 mmol) in toluene (20 mL) was added, then it was heated at 111 °C for 4 h. The reaction was cooled at 0 °C, crushed ice and saturated NH_4_Cl were added and then it was extracted with Et_2_O. The organic layer was washed with H_2_O and brine, dried over anhydrous Na_2_SO_4_, filtered and evaporated to yield **24** (8.7 g, 98%). IR (film, cm^−1^): 2916, 2849, 1471, 1385, 1152, 1103, 1010, 743, 695; ^1^H-NMR (200 MHz, CDCl_3_, δ ppm): 7.54–7.49 (2H, m, H-2′, 6′), 7.40–7.32 (3H, m, H-3′, 4′, 5′), 5.55 (1H, s, O–CH–O), 4.33 (2H, d, *J* = 12.6 Hz, H_A_-1, H_A_-3), 4.15 (1H, m, H-2), 4.06 (2H, d, *J* = 12.6 Hz, H_B_-1, H_B_-3), 3.55 (2H, t, *J* = 6.8 Hz, H-1′′), 1.65 (2H, m, H-2′′), 1.26 (30H, m, H-3′′-17′′), 0.88 (3H, t, *J* = 6.4 Hz, H-18′′); ^13^C-NMR (50 MHz, CDCl_3_, δ ppm): 138.5 (C-1′), 129.0 (C-4′), 128.5 (C-2′, 6′), 126.5 (C-3′, 5′), 101.5 (O–CH–O), 72.5 (C-1′′), 70.9 (C-2), 69.2 (C-1, 3), 32.2 (C-16′′), 30.1–29.2 (C-2′′, 4′′-15′′), 26.4 (C-3′′), 23.0 (C-17′′), 14.4 (C-18′′). EIHRMS: calcd. for C_28_H_48_O_3_ [M + Na]^+^: 455.3496, found: 455.3514.

### 4.13. Preparation of 2-O-Octadecylglycerol *(**25**)*

To a solution of **24** (8.7 g, 20 mmol) in MeOH (40 mL), *p*-TsOH (3.8 g, 20 mmol) was added and it was stirred at 35–40 °C for 6 h. Then H_2_O was added, the mixture was extracted with Et_2_O and washed with 6% NaHCO_3_ and H_2_O. The organic layer was dried over Na_2_SO_4_, filtered and evaporated. The residue was purified by column chromatography (EtOAc) to yield **25** (6.2 g, 90%). IR (film, cm^−1^): 3326, 2918, 2850, 1468, 1114, 1078, 1058, 975, 718; ^1^H-NMR (400 MHz, CDCl_3_, δ ppm): 3.78–3.66 (4H, m, H-1, H-3), 3.57 (2H, t, *J* = 6.7 Hz, H-1′′), 3.46 (1H, quin, *J* = 4.6 Hz, H-2), 1.59 (2H, m, H-2′′), 1.25 (30H, m, H-3′′-17′′), 0.88 (3H, t, *J* = 6.6 Hz, H-18′′); ^13^C-NMR (50 MHz, CDCl_3_, δ ppm): 79.9 (C-2), 70.4 (C-1′′), 62.1 (C-1), 62.1 (C-3), 32.1 (C-16′′), 30.2 (C-2′′), 29.7 (C-4′′-15′′), 26.3 (C-3′′), 22.9 (C-17′′), 14.3 (C-18′′). EIHRMS: calcd. for C_21_H_44_O_3_ [M + Na]^+^: 367.3183, found: 367.3187.

### 4.14. Reaction of **25** with TBDMSCl: Preparation of 1-O-tert-Butyldimethylsilyl-2-O-octadecyl-glycerol *(**26**)* and 1,3-O-di-tert-Butyldimethylsilyl-2-O-octadecylglycerol *(**27**)*:

To an ice-cooled solution of **25** (3.4 g, 9.9 mmol) in DMF (99 mL), TBDMSCl (1.49 g, 9.9 mmol) and imidazole (673 mg, 9.9 mmol) were added. It was stirred overnight at rt under an argon atmosphere; the reaction mixture was cooled at 0 °C and quenched with H_2_O. It was extracted with Et_2_O and the organic layer washed with H_2_O. After drying over anhydrous Na_2_SO_4_ the solvent was evaporated. The crude purified by column chromatography (Hex/EtOAc 97:3) to give **26** (1.85 g, 41%); **27** (910 mg, 16%) and **25** (1.43 g, 42%).

*Compound* **26**: IR (film, cm^−1^): 3450, 2925, 2854, 1475, 1100, 837; ^1^H-NMR (400 MHz, CDCl_3_, δ ppm): 3.73 (1H, dd, *J* = 10.2, 4.0 Hz, H_A_-1), 3.72 (1H, dd, *J* = 12.0, 5.2 Hz, H_A_-3), 3.62 (1H, dd, *J* = 12.0, 5.2 Hz, H_B_-3), 3.61 (1H, dd, *J* = 10.2, 6.8 Hz, H_B_-1), 3.58 (1H, ddd, *J* = 9.2, 6.8, 6.8 Hz, H_A_-1′′), 3.52 (1H, ddd, *J* = 9.2, 6.8, 6.8 Hz, H_B_-1′′), 3.42 (1H, dddd, *J* = 6.8, 5.2, 5.2, 4.0 Hz, H-2), 2.15 (1H, m,–OH), 1.55 (2H, m, H-2′′), 1.25 (30H, m, H-3′′-17′′), 0.89 (9H, s, Me_3_CSi-), 0.89 (3H, t, *J* = 6.8 Hz, H-18′′), 0.06 (6H, s, Me_2_Si-); ^13^C-NMR (100 MHz, CDCl_3_, δ ppm):80.1 (C-2), 70.7 (C-1′′), 63.2 (C-3), 62.9 (C-1), 32.1 (C-16′′), 30.3 (C-2′′), 29.7 (C-4′′-15′′), 26.3 (C-3′′), 26.0 (Me_3_CSi-), 22.9 (C-17′′), 18.4 (Me_3_CSi-), 14.3 (C-18′′), −5.3 (Me_2_Si-). EIHRMS: calcd. for C_27_H_58_O_3_Si [M + Na]^+^: 481.4047, found: 481.4025.

*Compound* **27**: IR (film, cm^−1^): 2926, 2855, 1475, 1257, 1106, 836; ^1^H-NMR (200 MHz, CDCl_3_, δ ppm): 3.72–3.53 (4H, m, H-1, H-3), 3.56 (2H, t, *J* = 6.6 Hz, H-1′′), 3.34 (1H, quin, *J* = 5.4 Hz, H-2), 1.55 (2H, m, H-2′′), 1.26 (30H, m, H-3′′-17′′), 0.89 (2·9H, s, Me_3_CSi-), 0.89 (3H, t, *J* = 6.8 Hz, H-18′′), 0.05 (2·6H, s, Me_2_Si-); ^13^C-NMR (50 MHz, CDCl_3_, δ ppm): 81.4 (C-2), 70.9 (C-1′′), 63.1 (C-3), 63.1 (C-1), 32.2 (C-16′′), 30.4 (C-2′′), 29.9–29.6 (C-4′′-15′′), 26.4 (C-3′′), 26.1 (2·Me_3_CSi-), 22.9 (C-17′′), 18.5 (2·Me_3_CSi-), 14.3 (C-18′′), −5.2 (2·Me_2_Si-). EIHRMS: calcd. for C_33_H_72_O_3_Si_2_ [M + Na]^+^: 595.4912, found: 595.4927.

### 4.15. Preparation of 1-Chlorocarbonyl-2-O-octadecyl-3-O-tert-butyldimethylsilylglycerol *(**28**)*

To an ice cooled solution of **26** (500 mg, 1 mmol) in THF (2 mL), trichloromethyl chloroformate (diphosgene, 0.12 mL, 1 mmol) and *N*,*N*-dimethylaniline (0.13 mL, 1 mmol) were slowly added. The mixture was stirred at 0 °C for 10 min and then at rt for 4 h. Then Et_2_O was added and the solution washed with 0.2 M HCl, 0.2 M NaOH and H_2_O, dried over anhydrous Na_2_SO_4_ and evaporated. The reaction bulk was purified by column chromatography to separate **28** (370 mg, 71%). IR (film, cm^−1^): 2925, 2854, 1781, 1462, 1254, 1164, 838, 780; ^1^H-NMR (200 MHz, CDCl_3_, δ ppm): 4.49 (1H, dd, *J* = 11.4, 3.4 Hz, H_A_-1), 4.33 (1H, dd, *J* = 11.4, 5.0 Hz, H_B_-1), 3.83–3.60 (2H, m, H-3), 3.60–3.50 (1H, m, H-2), 3.54 (2H, t, *J* = 6.6 Hz, H-1′′), 1.55 (2H, m, H-2′′), 1.26 (30H, m, H-3′′-17′′), 0.89 (9H, s, Me_3_CSi-), 0.88 (3H, t, *J* = 6.8 Hz, H-18′′), 0.06 (6H, s, Me_2_Si-); ^13^C-NMR (50 MHz, CDCl_3_, δ ppm): 150.9 (O–CO–Cl), 77.6 (C-2), 71.2 (C-1′′), 71.0 (C-1), 61.9 (C-3), 32.2 (C-16′′), 30.3 (C-2′′), 29.9–29.6 (C-4′′-15′′), 26.2 (C-3′′), 26.0 (Me_3_CSi-), 22.9 (C-17′′), 18.4 (Me_3_CSi-), 14.3 (C-18′′), −5.3 (Me_2_Si-). EIHRMS: in MeOH, calcd. for the methyl ester, C_29_H_60_O_5_Si [M + Na]^+^: 539.4102, found: 539.4122.

### 4.16. Preparation of 1-O-[1,25-Epoxy-18-nor-ent-isodysidiola-1,3(25),9,19-tetraen-4R/S-yloxycarbonyl]-2-O-octadecyl-3-O-tert-butyldimethylsilylglycerol *(**29**)*

To a solution of **1**/**2** (324 mg, 0.91 mmol), *N*,*N*-diisopropylethylamine (DIPEA, 0.22 mL, 1.27 mmol), 4-(dimethylamino) pyridine (DMAP, 56 mg, 0.46 mmol) in toluene (4.6 mL), a solution of **28** (364 mg, 0.7 mmol) in toluene (3.5 mL) was added dropwise at 0 °C . The reaction mixture was stirred at 0 °C under an argon atmosphere for 15 min and then at rt overnight. After this time the solvent was removed and the residue was purified by column chromatography (Hex/Et_2_O 99.9:0.1) to obtain **29** (330 mg, 56%). IR (film, cm^−1^): 2926, 2855, 1745, 1464, 1256, 1107, 837, 777; ^1^H-NMR (400 MHz, CDCl_3_, δ ppm): 7.43/7.42 (1H, s, H-25′), 7.33 (1H, s, H-1′), 6.39 (1H, s, H-2′), 5.79–5.76 (1H, m, H-4′), 5.35–5.32 (1H, m, H-9′), 4.66–4.60 (2H, m, H-20′), 4.32–4.06 (2H, m, H-1), 3.64–3.57 (2H, m, H-3), 3.54–3.49 (1H, m, H-2), 3.54–3.49 (2H, m, H-1′′), 2.20–1.40 (16H, m, H-5′, 7′, 8′, 11′, 12′, 13′, 14′, 16′, 17′), 1.70/1.66 (3H, s, H-21′), 1.54 (2H, m, H-2′′), 1.25 (30H, m, H-3′′-17′′), 0.89 (3H, s, H-22′), 0.89 (3H, s, H-24′), 0.88 (3H, t, *J* = 6.8 Hz, H-18′′), 0.87 (9H, s, Me_3_CSi-), 0.81 (3H, d, *J* = 6.9 Hz, H-23′), 0.04 (6H, s, Me_2_Si-); ^13^C-NMR (50 MHz, CDCl_3_, δ ppm): 154.6/154.5 (O–CO–O), 147.0 (C-19′), 143.2/143.1 (C-1′), 142.6 (C-10′), 140.1 (C-25′), 126.1 (C-3′), 120.0/119.7 (C-9′), 109.1/109.0 (C-20′), 108.7/108.6 (C-2′), 78.0 (C-2), 70.7 (C-1′′), 70.0/69.9 (C-4′), 67.1/66.8 (C-1), 62.2 (C-3), 44.2 (C-5′), 42.9 (C-15′), 42.4 (C-11′), 38.8 (C-14′), 37.4 (C-16′), 34.1/34.0 (C-6′), 32.4/32.3 (C-17′), 31.9 (C-16′′), 30.0–29.3 (C-2′′, 4′′-15′′, 7′, 13′), 26.0 (C-3′′), 25.8 (Me_3_CSi-), 22.8 (C-12′), 22.6 (C-8′), 22.6 (C-17′′), 22.4 (C-24′), 22.2 (C-21′), 22.2 (C-22′), 18.2 (Me_3_CSi-), 15.6 (C-23′), 14.1 (C-18′′), −5.2 (Me_2_Si-). EIHRMS: calcd. for C_52_H_92_O_6_Si [M + Na]^+^: 863.6555, found: 863.6542.

### 4.17. Preparation of 1-O-[1,25-Epoxy-18-nor-ent-isodysidiola-1,3(25),9,19-tetraen-4R/S-yloxycarbonyl]-2-O-octadecylglycerol *(**30**)*

To a solution of **29** (126 mg, 0.15 mmol) in THF (1.7 mL), 1 M TBAF in THF (0.22 mL, 0.22 mmol) was added under an argon atmosphere. The mixture was stirred for 2 h at rt, then the reaction was quenched with H_2_O and extracted with EtOAc. The organic layer was washed with H_2_O and brine, dried over anhydrous Na_2_SO_4_, filtered and removed the solvent. The residue was purified by column chromatography (EtOAc) providing **30** (97 mg, 89%). IR (film, cm^−1^): 3335, 2922, 2853, 1744, 1466, 1258, 1078, 1059, 1024; ^1^H-NMR (400 MHz, CDCl_3_, δ ppm): 7.42 (1H, s, H-25′), 7.34 (1H, s, H-1′), 6.39 (1H, s, H-2′), 5.78 (1H, m, H-4′), 5.35–5.32 (1H, m, H-9′), 4.66–4.61 (2H, m, H-20′), 4.22–4.14 (2H, m, H-1), 3.75–3.67 (2H, m, H-3), 3.56 (2H, t, *J* = 6.4 Hz, H-1′′), 3.49–3.43 (1H, m, H-2), 2.20–1.40 (16H, m, H-5′, 7′, 8′, 11′, 12′, 13′, 14′, 16′, 17′), 1.69/1.66 (3H, s, H-21′), 1.55 (2H, m, H-2′′), 1.25 (30H, m, H-3′′-17′′), 0.89 (3H, s, H-22′), 0.88 (3H, t, *J* = 6.8 Hz, H-18′′), 0.87 (3H, s, H-24′), 0.80/0.79 (3H, d, *J* = 6.8 Hz, H-23′); ^13^C-NMR (50 MHz, CDCl_3_, δ ppm): 154.8 (O–CO–O), 147.3 (C-19′), 143.5 (C-1′), 141.6/141.3 (C-10′), 140.4 (C-25′), 126.2 (C-3′), 120.2/120.0 (C-9′), 109.4/109.3 (C-20′), 108.9/108.8 (C-2′), 79.8 (C-2), 70.9/70.4 (C-1′′), 70.5 (C-4′), 66.5/66.4 (C-1), 62.4/62.1 (C-3), 44.4 (C-5′), 43.1 (C-15′), 42.7/42.6 (C-11′), 39.1 (C-14′), 37.6/37.4 (C-16′), 34.4/34.3 (C-6′), 32.7/32.6 (C-17′), 32.1 (C-16′′), 31.5 (C-7′), 30.3–29.6 (C-2′′, 4′′-15′′), 29.1 (C-13′), 26.3/26.2 (C-3′′), 23.4 (C-24′), 23.1 (C-21′), 22.9 (C-8′), 22.9 (C-12′), 22.9 (C-17′′), 22.5 (C-22′), 15.9 (C-23′), 14.3 (C-18′′). EIHRMS: calcd. for C_46_H_78_O_6_ [M + Na]^+^: 749.5691, found: 749.5665.

### 4.18. Preparation of 1-O-[1,25-Epoxy-18-nor-ent-isodysidiola-1,3(25),9,19-tetraen-4R/S-yloxycarbonyl]-2-O-octadecyl-glycero-3-phosphate *(**31**)*

To a solution of **30** (37 mg, 0.05 mmol) and anhydrous pyridine (8 μL) in THF (0.3 mL), POCl_3_ (5 μL, 0.06 mmol) was added dropwise under an argon atmosphere with stirring at 0 °C for 5 h. Then 6% NaHCO_3_ was added and the mixture stirred for an additional 15 min. at 0 °C. After that time, crushed ice was added, the mixture was acidified with 2 M HCl to pH = 2 and extracted with EtOAc_._ The organic layer was washed with H_2_O and the solvent removed to give **31** (39 mg, 97%). IR (film, cm^−1^): 2924, 2853, 1744, 1466, 1258 ^1^H-NMR (200 MHz, CDCl_3_, δ ppm): 7.39 (1H, broad s, H-25′), 7.3 (1H, broad s, H-1′), 6.37 (1H, broad s, H-2′), 5.76 (1H, m, H-4′), 5.33 (1H, m, H-9′), 4.64–4.61 (2H, m, H-20′), 4.34–3.83 (4H, m, H-1, H-3), 3.64–3.31 (3H, m, , H-1′′, H-2), 2.10–1.40 (16H, m, H-5′, 7′, 8′, 11′, 12′, 13′, 14′, 16′, 17′), 1.69/1.65 (3H, s, H-21′), 1.55 (2H, m, H-2′′), 1.25 (30H, m, H-3′′-17′′), 0.89 (3H, s, H-22′), 0.88 (3H, t, *J* = 6.8 Hz, H-18′′), 0.87 (3H, s, H-24′), 0.80/0.79 (3H, d, *J* = 6.8 Hz, H-23′).

### 4.19. Preparation of 1-O-[1,25-Epoxy-18-nor-ent-isodysidiola-1,3(25),9,19-tetraen-4R/S-yloxycarbonyl]-2-O-octadecyl-glycero-3-phosphocoline *(**9**)*

Compound **31** (39 mg, 0.05 mmol), choline tetraphenyl borate (21 mg, 0.05 mmol) and TPS (18 mg, 0.06 mmol) were dissolved in anhydrous pyridine (0.4 mL). The mixture was heated at 70 °C for 1 h and then it was stirred at rt overnight. After addition of H_2_O (0.1 mL), the solvents were removed by rotary evaporation. The crude mixture was dissolved in Et_2_O and stirred for a few minutes. The solid formed was eliminated by filtration. The organic solution was evaporated and the reaction bulk purified by column chromatography (CHCl_3_/MeOH/NH_4_OH 65:30:5) to yield **9** (16 mg, 35%). IR (film, cm^−1^): 2920, 2851, 1738, 1467; ^1^H-NMR (400 MHz, CDCl_3_, δ ppm):7.42 (1H, s, H-25′), 7.37 (1H, s, H-1′), 6.39 (1H, s, H-2′), 5.75 (1H, m, H-4′), 5.36–5.34 (1H, m, H-9′), 4.66–4.61 (2H, m, H-20′), 4.37 (2H, m, H-1′′′), 4.33–4.10 (2H, m, H-1), 4.00–3.80 (2H, m, H-3), 3.98–3.75 (2H, m, H-2′′′), 3.65 (1H, m, H-2), 3.51 (2H, m, H-1′′), 3.35 (9H, s, Me_3_N–), 2.05–1.40 (16H, m, H-5′, 7′, 8′, 11′, 12′, 13′, 14′, 16′, 17′), 1.70/1.67 (3H, s, H-21′), 1.54 (2H, m, H-2′′), 1.26 (30H, m, H-3′′-17′′), 0.90 (3H, s, H-22′), 0.88 (3H, t, *J* = 6.8 Hz, H-18′′), 0.87 (3H, s, H-24′), 0.82/0.81 (3H, d, *J* = 6.9 Hz, H-23′); ^13^C-NMR (100 MHz, CDCl_3_, δ ppm): 154.5 (O–CO–O), 146.9 (C-19′), 143.3 (C-1′), 141.4/141.0 (C-10′), 140.0 (C-25′), 126.2 (C-3′), 120.0/119.7 (C-9′), 109.1/109.0 (C-20′), 108.7 (C-2′), 76.2 (C-2), 70.8/70.6 (C-1′′), 70.2 (C-4′), 66.9 (C-1), 66.2 (C-2′′′), 64.4 (C-3), 59.7 (C-1′′′), 54.4 (Me_3_N-), 44.3 (C-5′), 42.9 (C-15′), 42.6/42.5 (C-11′), 38.8/38.7 (C-14′), 37.3/37.2 (C-16′), 34.1 (C-6′), 32.4/32.3 (C-17′), 31.9 (C-16′′), 31.2 (C-7′), 29.9–29.3 (C-2′′, 4′′-15′′), 28.9/28.8 (C-13′), 25.9 (C-3′′), 23.2 (C-12′), 22.8 (C-24′), 22.7 (C-21′), 22.6 (C-8′), 22.6 (C-17′′), 22.2 (C-22′), 15.6 (C-23′), 14.0 (C-18′′). EIHRMS: calcd. for C_51_H_90_NO_9_P [M + Na]^+^: 914.6245, found: 914.6229.

### 4.20. Preparation of 1-O-[25-Hydroxy-18-nor-ent-isodysidiola-2,9,19-trien-1,25-olide-4R/S-yloxycarbonyl]-2-O-octadecyl-glycero-3-phosphocoline *(**10**)*

Rose Bengal (4 mg) was added to a solution of **9** (20 mg, 0.02 mmol) and DIPEA (38 μL, 0.22 mmol) in dry CH_2_Cl_2_ (10 mL) at rt. Anhydrous oxygen was bubbled in for 10 min and the solution was placed under an oxygen atmosphere at −78 °C and irradiated with a 200 W lamp. After 4 h irradiation was stopped, the pink solution allowed to warm to rt, and saturated aqueous oxalic acid solution (1.7 mL) added. After 30 min of vigorous stirring, the mixture was diluted with H_2_O and extracted with Et_2_O. The combined organic extracts were washed with H_2_O and brine. The solvent was evaporated to give a residue which was purified by silica gel column chromatography (CHCl_3_/MeOH/H_2_O 65:35:1) to yield **10** (6 mg, 30%). ^1^H-NMR (200 MHz, CDCl_3_, δ ppm): 6.28/6.10 (1H, s, H-25′), 5.91/5.83 (1H, m, H-2′), 5.65 (1H, m, H-4′), 5.36 (1H, m, H-9′), 4.63 (2H, m, H-20′), 4.33 (2H, m, H-1′′′), 4.32–4.10 (2H, m, H-1), 4.05–3.95 (2H, m, H-3), 4.00–3.80 (2H, m, H-2′′′), 3.65–3.55 (1H, m, H-2), 3.51 (2H, m, H-1′′), 3.34 (9H, s, Me_3_N–), 2.05–1.40 (16H, m, H-5′, 7′, 8′, 11′, 12′, 13′, 14′, 16′, 17′), 1.69 (3H, s, H-21′), 1.54 (2H, m, H-2′′), 1.25 (30H, m, H-3′′-17′′), 0.87 (6H, s, H-22′, 24′), 0.88 (3H, t, *J* = 7.0 Hz, H-18′′), 0.81 (3H, d, *J* = 6.9 Hz, H-23′); EIMS found for C_51_H_90_ NO_11_P [M + Na]^+^: 946.7.

### 4.21. Preparation of 1-O-[1,25-Epoxy-18-nor-ent-isodysidiola-1,3(25),9,19-tetraen-4R/S-yloxycarbonyl]-2-O-octadecyl-3-eicosa-pentaenoylglycerol *(**32**)*

To a solution of **30** (23 mg, 0.03 mmol), DMAP (5 mg, 0.04 mmol) and EDAC (8 mg, 0.04 mmol) in dry CH_2_Cl_2_ (0.3 mL), EPA (9.6 μL, 0.03 mmol) was added under an argon atmosphere. After stirring at rt for 13 h, the reaction mixture was passed through a short silica gel column (CH_2_Cl_2_/EtOAc 9:1 as eluent). Then the solvent was removed and the crude was purified by column chromatography (Hex/EtOAc 98:2) providing **32** (13 mg, 64%). IR (film, cm^−1^): 2959, 2926, 1740, 1560, 1383, 1261; ^1^H-NMR (400 MHz, CDCl_3_, δ ppm): 7.43/7.42 (1H, s, H-25′), 7.35/7.34 (1H, s, H-1′), 6.39 (1H, s, H-2′), 5.77 (1H, m, H-4′), 5.42–5.30 (10H, m, =CH), 5.42–5.30 (1H, m, H-9′), 4.64–4.61 (2H, m, H-20′), 4.21–4.07 (2H, m, H-1), 4.21–4.07 (2H, m, H-3), 3.67 (1H, m, H-2), 3.57–3.49 (2H, m, H-1′′), 2.84–2.80 (8H, m, =CCH_2_C=), 2.35–2.30 (2H, m, H-2′′′), 2.14–2.04 (4H, m, H-4′′′, H-19′′′), 2.00–1.40 (16H, m, H-5′, 7′, 8′, 11′, 12′, 13′, 14′, 16′, 17′), 1.74 (2H, m, H-3′′′), 1.70/1.67 (3H, s, H-21′), 1.54 (2H, m, H-2′′), 1.25 (30H, m, H-3′′-17′′), 0.97 (3H, t, *J* = 7.5 Hz, H-20′′′), 0.90 (3H, s, H-22′), 0.88 (3H, t, *J* = 7.0 Hz, H-18′′), 0.88 (3H, s, H-24′), 0.81/0.80 (3H, d, *J* = 6.9 Hz, H-23′); ^13^C-NMR (100 MHz, CDCl_3_, δ ppm): 173.1 (C-1′′′), 154.4 (O–CO–O), 147.0 (C-19′), 143.2 (C-1′), 141.0 (C-10′), 140.1 (C-25′), 132.0 (C-18′′′), 128.8–127.0 (=CH) × 9, 126.0 (C-3′), 120.0 (C-9′), 109.1/109.0 (C-20′), 108.7/108.6 (C-2′), 75.1/75.0 (C-2), 70.8/70.7 (C-1′′), 70.2 (C-4′), 66.7/66.4 (C-1), 63.0/62.8 (C-3), 44.2 (C-5′), 42.9 (C-15′), 42.5/42.3 (C-11′), 38.8 (C-14′), 37.3/37.2 (C-16′), 34.1/34.0 (C-6′), 33.5 (C-2′′′), 32.4/32.3 (C-17′), 31.9 (C-16′′), 31.2 (C-7′), 29.8–28.8 (C-2′′, 4′′-15′′), 29.9–29.8 (C-13′), 26.5–20.5 (3′′′, 4′′′, 7′′′, 10′′′, 13′′′, 16′′′, 19′′′), 25.9 (C-3′′), 22.8 (C-12′), 22.6 (C-8′), 22.6 (C-17′′), 22.3 (C-24′), 22.2 (C-21′), 22.2 (C-22′), 15.6 (C-23′), 14.2 (C-20′′′), 14.0 (C-18′′); EIHRMS: calcd. for C_66_H_106_O_7_ [M + Na]^+^: 1033.7831, found: 1033.7865.

### 4.22. Preparation of 1-O-[25-Hydroxy-18-nor-ent-isodysidiola-2,9,19-trien-1,25-olide-4R/S-yloxycarbonyl]-2-O-octadecyl-3-eicosapentaenoylglycerol *(**11**)*

Rose Bengal (1 mg) was added to a solution of **32** (9 mg, 0.009 mmol) and DIPEA (16 μL, 0.09 mmol) in dry CH_2_Cl_2_ (0.7 mL) at rt. Anhydrous oxygen was bubbled in for 10 min, the solution placed under oxygen atmosphere at −78 °C and irradiated with a 200 W lamp. After 4 h irradiation was stopped, the pink solution allowed to warm to rt, and saturated aqueous oxalic acid solution (0.7 mL) added. After 30 min of vigorous stirring, the mixture was diluted with H_2_O and extracted with CH_2_Cl_2_. The combined organic extracts were washed with H_2_O and brine, and dried over anhydrous Na_2_SO_4_. The solvent was evaporated to give a residue that was purified by silica gel column chromatography (Hex/EtOAc 9:1) to yield **11** (5 mg, 53%). IR (film, cm^−1^): 3387, 2924, 2855, 1747, 1454, 1258, 1134; ^1^H-NMR (400 MHz, CDCl_3_, δ ppm): 6.21/5.96 (1H, m, H-25′), 6.03/6.02 (1H, s, H-2′), 5.63 (1H, m, H-4′), 5.38–5.36 (10H, m, =CH), 5.38–5.36 (1H, m, H-9′), 4.67–4.61 (2H, m, H-20′), 4.33–4.06 (2H, m, H-1), 4.33–4.06 (2H, m, H-3), 3.70–3.67 (1H, m, H-2), 3.52 (2H, t, *J* = 6.7 Hz, H-1′′), 2.84–2.78 (8H, m, =CCH_2_C=), 2.34 (2H, t, *J* = 7.5 Hz, H-2′′′), 2.14–2.04 (4H, m, H-4′′′, H-19′′′), 2.00–1.40 (16H, m, H-5′, 7′, 8′, 11′, 12′, 13′, 14′, 16′, 17′), 1.74 (2H, m, H-3′′′), 1.70/1.69 (3H, s, H-21′), 1.54 (2H, m, H-2′′), 1.25 (30H, m, H-3′′-17′′), 0.97 (3H, t, *J* = 7.5 Hz, H-20′′′), 0.91 (3H, s, H-22′), 0.87 (3H, t, *J* = 6.8 Hz, H-18′′), 0.88 (3H, s, H-24′), 0.81 (3H, d, *J* = 6.9 Hz, H-23′); ^13^C-NMR (100 MHz, CDCl_3_, δ ppm): 173.4 (C-1′′′), 169.3 (C-1′), 168.2 (C-3′), 154.2 (O–CO–O), 147.2 (C-19′), 141.0 (C-10′), 132.0 (C-18′′′), 129.0–127.0 (=CH) × 9, 119.4 (C-9′), 118.4 (C-2′), 109.3/109.2 (C-20′), 97.4 (C-25′), 75.0 (C-2), 70.9 (C-1′′), 70.8 (C-4′), 66.9 (C-1), 62.3 (C-3), 43.1 (C-5′), 42.8 (C-15′), 42.6 (C-11′), 38.7 (C-14′), 37.4 (C-16′), 34.6 (C-6′), 33.5 (C-2′′′), 32.4 (C-17′), 31.9 (C-16′′), 29.6–29.4 (C-2′′, 4′′-15′′), 28.9 (C-13′), 29.3 (C-7′), 26.5–20.5 (3′′′, 4′′′, 7′′′, 10′′′, 13′′′, 16′′′, 19′′′), 25.9 (C-3′′), 22.9 (C-12′), 22.9 (C-8′), 22.8 (C-24′), 22.7 (C-21′),22.6 (C-17′′), 22.5 (C-22′), 15.6 (C-23′), 14.2 (C-20′′′), 14.1 (C-18′′). EIMS found for C_66_H_106_O_9_ [M + Na]^+^: 1065.7.

### 4.23. Preparation of 3-O-p-Methoxybenzyl-sn-glycerol *(**35**)*

To an ice cooled solution of (*S*)-(+)-solketal **33** (2.5 g, 18.9 mmol) in THF (94 mL), 60% NaH (756 mg, 31.5 mmol) and PMBCl (2.56 mL, 18.9 mmol) were added. The mixture was stirred at 0 °C for 10 min and at rt for 1 h. Then it was refluxed overnight, cooled to rt, and crushed ice and saturated NH_4_Cl added. The aqueous layer was extracted with EtOAc and the organic layer was washed with H_2_O and brine, dried over anhydrous Na_2_SO_4_, filtered and evaporated. The bulk reaction mixture was purified by column chromatography on silica gel (EtOAc) to obtain **35** (3.6 g, 90%). ${\left[\alpha \right]}_{D}^{22}$ −2.43 (*c* 0.7, CHCl_3_); IR (film, cm^−1^): 3395, 2934, 2866, 1612, 1514, 1248, 1082, 1034; ^1^H-NMR (400 MHz, CDCl_3_, δ ppm): 7.25 (2H, d, *J* = 7.2 Hz, H-2′, H-6′), 6.89 (2H, d, *J* = 8.6 Hz, H-3′, H-5′), 4.48 (2H, s, –OCH_2_Ar), 3.88 (1H, m, H-*sn*2), 3.81 (3H, s, –OCH_3_), 3.70 (1H, dd, *J* = 11.4, 3.9 Hz, H_A_-*sn*1), 3.63 (1H, dd, *J* = 11.4, 5.3 Hz, H_B_-*sn*1), 3.56 (1H, dd, *J* = 9.6, 3.9 Hz, H_A_-*sn*3), 3.52 (1H, dd, *J* = 9.6, 6.2 Hz, H_B_-*sn*3); ^13^C-NMR (50 MHz, CDCl_3_, δ ppm): 159.6 (C-4′′′), 130.0 (C-1′′′), 129.7 (C-2′′′, C-6′′′), 114.1 (C-3′′′, C-5′′′), 73.4 (–OCH_2_Ar), 71.6 (C-*sn*3), 71.0 (C-*sn*2), 64.2 (C-*sn*1), 55.5 (–OCH_3_). EIHRMS: calcd. for C_11_H_16_O_4_ [M + Na]^+^: 235.0941, found: 235.0946.

### 4.24. Preparation of 3-O-p-Methoxybenzyl-1-O-trityl-sn-glycerol *(**36**)*

To a solution of **35** (2.4 g, 11 mmol) in pyridine (23 mL), TrCl (3.1 g, 11 mmol) was added and the mixture was heated to boiling for 15 h. The reaction mixture was allowed to cool to rt and H_2_O was added, then it was extracted with EtOAc and washed with 2 M HCl, 6% NaHCO_3_ and brine, dried over anhydrous Na_2_SO_4_ and filtered. The removal of the solvent led to a crude which was purified by column chromatography (Hex/EtOAc 9:1) to obtain **36** (4.6 g, 92%). ${\left[\alpha \right]}_{D}^{22}$ −0.7 (*c* 0.8, CHCl_3_); IR (film, cm^−1^) 3449, 2932, 2870, 1512, 1491, 1448, 1248, 1076, 1034, 765, 706, 633; ^1^H-NMR (400 MHz, CDCl_3_) δ 7.46–7.42 (6H, m, H-2′, 6′), 7.32–7.24 (9H, m, H-3′-5′), 7.22 (2H, d, *J* = 8.6 Hz, H-2′′′, H-6′′′), 6.87 (2H, d, *J* = 8.6 Hz, H-3′′′, H-5′′′), 4.48 (2H, s, –OCH_2_Ar), 3.99 (1H, m, H-*sn*2), 3.81 (3H, s, –OCH_3_), 3.59 (1H, dd, *J* = 9.7, 4.3 Hz, H_A_-*sn*3), 3.54 (1H, dd, *J* = 9.7, 6.2 Hz, H_B_-*sn*3) 3.25 (1H, dd, *J* = 9.4, 5.7 Hz, H_A_-*sn*1), 3.21 (1H, dd, *J* = 9.4, 5.7 Hz, H_B_-*sn*1); ^13^C-NMR (100 MHz, CDCl_3_) δ 159.2 (C-4′′′), 143.8 (C-1′), 130.1 (C-1′′′), 129.3 (C-2′′′, C-6′′′), 128.6 (C-3′, C-5′), 127.8 (C-2′, C-6′), 127.0 (C-4′), 113.8 (C-3′′′, C-5′′′), 86.6 (–CPh_3_), 73.0 (–OCH_2_Ar), 71.2 (C-*sn*3), 69.9 (C-*sn*2), 64.4 (C-*sn*1), 55.2 (–OCH_3_). EIHRMS: calcd. for C_30_H_30_O_4_ [M + Na]^+^ 477.2036, found [M + Na]^+^ 477.2022.

### 4.25. Preparation of 3-O-p-Methoxybenzyl-2-O-octadecyl-1-O-trityl-sn-glycerol *(**37**)*

To a solution of **36** (570 mg, 1.26 mmol) in toluene (2.5 mL), NaNH_2_ (491 mg, 12.6 mmol) was added, it was heated at 111 °C under an argon atmosphere for 1 h. The mixture was cooled to rt and a solution of bromooctadecane (1.7 g, 5 mmol) in toluene (2 mL) added, then heated at 111 °C overnight. The reaction was allowed to cool to 0 °C, crushed ice and saturated NH_4_Cl added and extracted with EtOAc. The organic layer was washed with H_2_O and brine. After drying over anhydrous Na_2_SO_4_, filtering and evaporating, the reaction mixture was purified by column chromatography (Hex/EtOAc 98:2) to yield **37** (865 mg, 97%). ${\left[\alpha \right]}_{D}^{22}$ −3.6 (c 1.2, CHCl_3_); ^1^H-NMR (400 MHz, CDCl_3_, δ ppm): 7.47–7.44 (6H, m, H-2′, 6′), 7.31–7.24 (9H, m, H-3′-5′), 7.19 (1H, d, each, *J* = 8.6 Hz, H-2′′′, H-6′′′), 6.84 (1H, d, each, *J* = 8.6 Hz, H-3′′′, H-5′′′), 4.48, 4.43 (1H, d, each, *J* = 11.7 Hz, –OCH_2_Ar), 3.80 (3H, s, –OCH_3_), 3.61–3.54 (1H, m, H-*sn*2), 3.61–3.54 (2H, m, H-*sn*3), 3.53 (2H, t, *J* = 6.6 Hz, H-1′′), 3.21 (2H, d, *J* = 4.6 Hz, H-*sn*1), 1.56 (2H, m, H-2′′), 1.27 (30H, m, H-3′′-17′′), 0.89 (3H, t, *J* = 6.8 Hz, H-18′′); ^13^C-NMR (50 MHz, CDCl_3_, δ ppm): 159.3 (C-4′′′), 144.4 (3·C-1′), 130.8 (C-1′′′), 129.4 (C-2′′′, C-6′′′), 129.0/128.2 (3·C-3′, 3·C-5′), 128.0/127.5 (3·C-2′, 3·C-6′), 127.1 (3·C-4′), 113.9 (C-3′′′, C-5′′′), 86.8 (–CPh_3_), 78.6 (C-*sn*2), 73.1 (–OCH_2_Ar), 70.9 (C-1′′), 70.4 (C-*sn*3), 63.7 (C-*sn*1), 55.5 (–OCH_3_), 32.2 (C-16′′), 30.4–29.6 (C-2′′, 4′′-15′′), 26.4 (C-3′′), 22.9 (C-17′′), 14.4 (C-18′′); EIHRMS: calcd. for C_48_H_66_O_4_ [M + Na]^+^: 729.4853, found: 729.4854.

### 4.26. Preparation of 3-O-p-Methoxybenzyl-2-O-octadecyl-sn-glycerol *(**38**)*

To a mixture of **37** (865 mg, 1.22 mmol) in MeOH (12 mL) and CHCl_3_ (1 mL), *p*-TsOH (232 mg, 1.22 mmol) was added, and the mixture was stirred under an argon atmosphere at rt for 2 h 30 min., H_2_O added and the mixture extracted with EtOAc. The organic layer washed with 6% NaHCO_3_ and H_2_O, dried over anhydrous Na_2_SO_4_, filtered and evaporated. The residue was purified by column chromatography (Hex/EtOAc 95:5) to yield **38** (464 mg, 82%). ${\left[\alpha \right]}_{D}^{22}$ +8.0 (c 0.9, CHCl_3_); IR (film, cm^−1^): 3451, 2922, 2853, 1514, 1248, 1094, 1040; ^1^H-NMR (400 MHz, CDCl_3_, δ ppm): 7.25 (2H, d, *J* = 8.6 Hz, H-2′′′, H-6′′′), 6.88 (2H, d, *J* = 8.6 Hz, H-3′′′, H-5′′′), 4.49, 4.45 (1H, d, each, *J* = 11.9 Hz, –OCH_2_Ar), 3.80 (3H, s, –OCH_3_), 3.74–3.55 (2H, m, H-*sn*1), 3.55–3.47 (1H, m, H-*sn*2), 3.55–3.47 (2H, m, H-*sn*3), 3.53 (2H, t, *J* = 4.4 Hz, H-1′′), 1.56 (2H, m, H-2′′), 1.26 (30H, m, H-3′′-17′′), 0.88 (3H, t, *J* = 6.8 Hz, H-18′′); ^13^C-NMR (100 MHz, CDCl_3_, δ ppm): 159.5 (C-4′′′), 130.3 (C-1′′′), 129.5 (C-2′′′, C-6′′′), 114.0 (C-3′′′, C-5′′′), 78.6 (C-*sn*2), 73.4 (–OCH_2_Ar), 70.6 (C-1′′), 69.9 (C-*sn*3), 63.2 (C-*sn*1), 55.5 (–OCH_3_), 32.2 (C-16′′), 30.3–29.6 (C-2′′, 4′′-15′′), 26.3 (C-3′′), 22.9 (C-17′′), 14.4 (C-18′′); EIHRMS: calcd. for C_29_H_52_O_4_ [M + Na]^+^: 487.3758, found: 487.3755.

### 4.27. Preparation of 1-Chlorocarbonyl-3-O-p-methoxybenzyl-2-O-octadecyl-sn-glycerol *(**39**)*

To an ice cooled solution of **38** (261 mg, 0.56 mmol) in THF (1.1 mL), trichloromethyl chloroformate (diphosgene, 67 μL, 0.56 mmol) and *N*,*N*-dimethylaniline (71 μL, 0.56 mmol) were slowly added. The mixture was stirred at 0 °C for 15 min and then at rt for 2 h. Then Et_2_O was added and the white precipitate filtered. The solution washed with 0.2 M HCl, 0.2 M NaOH and H_2_O, dried over anhydrous Na_2_SO_4_ and evaporated to give **39** (244 mg, 82%). IR (film, cm^−1^): 2924, 2853, 1774, 1248, 1167, 1101; ^1^H-NMR (400 MHz, CDCl_3_, δ ppm): 7.24 (2H, d, *J* = 8.4 Hz, H-2′′′, H-6′′′), 6.88 (2H, d, *J* = 8.4 Hz, H-3′′′, H-5′′′), 4.47 (2H, s, –OCH_2_Ar), 4.45 (1H, dd, *J* = 11.2, 3.7 Hz, H_A_-*sn*1), 4.36 (1H, dd, *J* = 11.2, 6.2 Hz, H_B_-*sn*1), 3.80 (3H, s, –OCH_3_), 3.68 (1H, m, H-*sn*2), 3.56–3.52 (2H, m, H-1′′), 3.55–3.51 (1H, dd, *J* = 10.0, 4.7 Hz, H_A_-*sn*3), 3.49–3.45 (1H, dd, *J* = 10.0, 6.2 Hz, H_B_-*sn*3) 1.54 (2H, m, H-2′′), 1.26 (30H, m, H-3′′-17′′), 0.88 (3H, t, *J* = 6.8 Hz, H-18′′); ^13^C-NMR (100 MHz, CDCl_3_, δ ppm): 159.3 (C-4′′′), 150.7 (–O–CO–Cl), 129.8 (C-1′′′), 129.3 (C-2′′′, C-6′′′), 113.8 (C-3′′′, C-5′′′), 75.8 (C-*sn*2), 73.1 (–OCH_2_Ar), 71.1 (C-*sn*1), 70.8 (C-1′′), 68.2 (C-*sn*3), 55.2 (–OCH_3_), 31.9 (C-16′′), 29.8–29.3 (C-2′′, 4′′-15′′), 25.9 (C-3′′), 22.6 (C-17′′), 14.1 (C-18′′); EIHRMS: in MeOH, calcd. for methyl ester, C_31_H_54_O_6_ [M + Na]^+^: 545.3813, found: 545.3794.

### 4.28. Preparation of 1-O-[1,25-Epoxy-18-nor-ent-isodysidiola-1,3(25),9,19-tetraen-4R/S-yloxycarbonyl]-2-O-octadecyl-3-O-p-methoxybenzyl-sn-glycerol *(**40**)*

To a solution of **1**/**2** (176 mg, 0.49 mmol), *N*,*N*-diisopropylethylamine (DIPEA, 0.14 mL, 0.79 mmol), 4-(dimethylamino) pyridine (DMAP) (30 mg, 0.25 mmol) in toluene (2.5 mL), a solution of **39** (244 mg, 0.46 mmol) in toluene (2.3 mL) was added dropwise at 0 °C . The reaction mixture was stirred at 0 °C under an argon atmosphere for 15 min and then at rt for 20 h. Then the solvent was removed and the residue was purified by column chromatography (Hex/EtOAc 98:2) to obtain **40** (226 mg, 58%). ${\left[\alpha \right]}_{D}^{22}$ −7.1 (*c* 0.8, CHCl_3_); IR (film, cm^−1^): 2924, 2853, 1745, 1514, 1250; ^1^H-NMR (400 MHz, CDCl_3_, δ ppm): 7.42/7.41 (1H, broad s, H-25′), 7.34/7.33 (1H, broad s, H-1′), 7.24/7.22 (2H, d, *J* = 8.6 Hz, H-2′′′, H-6′′′), 6.87/6.86 (2H, d, *J* = 8.6 Hz, H-3′′′, H-5′′′), 6.39 (1H, s, H-2′), 5.79/5.77 (1H, dd, *J* = 5.4, 3.3 Hz, H-4′), 5.35/5.33 (1H, t, *J* = 4.0 Hz, H-9′), 4.65–4.60 (2H, m, H-20′), 4.47/4.44 (2H, s, –OCH_2_Ar), 4.38/4.37 (1H, dd, each, *J* = 11.2, 4.0 Hz, H_A_-*sn*1), 4.30–4.25/4.20–4.10 (1H, m, each, H_B_-*sn*1), 3.80 (3H, s, –OMe), 3.70–3.60 (1H, m, H-*sn*2), 3.56–3.41 (2H, m, H-*sn*3), 3.48 (2H, t, *J* = 5.0 Hz, H-1′′), 2.00–1.49 (16H, m, H-5′, 7′, 8′, 11′, 12′, 13′, 14′, 16′, 17′), 1.70/1.66 (3H, s, Me-21′), 1.56 (2H, m, H-2′′), 1.25 (30H, m, H-3′′-17′′), 0.91 (3H, s, Me-22′), 0.89 (3H, t, *J* = 7.0 Hz, Me-18′′), 0.88 (3H, s, Me-24′), 0.81/0.80 (3H, d, *J* = 7.0 Hz, Me-23′); ^13^C-NMR (50 MHz, CDCl_3_, δ ppm): 159.5/159.4 (C-4′′′), 154.8 (–O–CO–O–), 147.3 (C-19′), 143.5 (C-1′), 141.6/141.3 (C-10′), 140.3 (C-25′), 130.4/130.2 (C-1′′′), 129.5 (C-2′′′, 6′′′), 126.3 (C-3′), 120.3/120.0 (C-9′), 114.0 (C-3′′′, 5′′′), 109.4/109.3 (C-20′), 109.0/108.9 (C-2′), 76.6 (C-*sn*2), 73.3 (–OCH_2_Ar), 71.0 (C-*sn*1), 70.3 (C-4′), 69.3/69.0 (C-1′′), 68.5 (C-*sn*3), 55.5 (–OMe), 44.4 (C-5′), 43.1 (C-15′), 42.8/42.6 (C-11′), 39.1 (C-14′), 37.6/37.4 (C-16′), 34.4/34.3 (C-6′), 32.6 (C-17′), 32.2 (C-16′′), 30.2–29.0 (C-2′′), 29.6–28.9 (C-4′′-15′′), 29.6–28.9 (C-7′, 13′), 26.2/26.1 (C-3′′), 23.1 (C-8′, 12′), 24.2 (C-24′), 23.0 (C-21′), 22.9 (C-17′′), 22.5 (C-22′), 15.9 (C-23′), 14.4 (C-18′′); EIHRMS: calcd. for C_54_H_86_O_7_ [M + Na]^+^: 869.6266, found: 869.6260.

### 4.29. Preparation of 1-O-[1,25-Epoxy-18-nor-ent-isodysidiola-1,3(25),9,19-tetraen-4R/S-yloxycarbonyl]-2-O-octadecyl-sn-glycerol *(**41**)*

To a solution of **40** (167 mg, 0.20 mmol) in CH_2_Cl_2_/H_2_O 18:1 (2.2 mL), DDQ (10 mg, 0.045 mmol) was added. It was stirred at rt under an argon atmosphere for 7 h, then quenched with 6% NaHCO_3_ and extracted with CH_2_Cl_2._ The organic layer was washed with 6% NaHCO_3_ and brine and dried over anhydrous Na_2_SO_4_. Removal of the solvent gave a crude product which was purified by column chromatography (Hex/EtOAc 97:3) on silica gel to obtain **41** (103 mg, 71%). ${\left[\alpha \right]}_{D}^{22}$ +15.2 (*c* 1, CHCl_3_); IR (film, cm^−1^): 3506, 2924, 2853, 1746, 1258; ^1^H-NMR (400 MHz, CDCl_3_, δ ppm): 7.42 (1H, s, H-25′), 7.34 (1H, s, H-1′), 6.39 (1H, s, H-2′), 5.80/5.75 (1H, m, H-4′), 5.36/5.33 (1H, t, *J* = 3.4 Hz, H-9′), 4.65–4.61 (2H, m, H-20′), 4.21/4.20 (1H, dd, each, *J* = 11.4, 4.8 Hz, H_A_-*sn*3), 4.16/4.13 (1H, dd, each, *J* = 11.4, 4.8 Hz, H_B_-*sn*3) 3.68–3.46 (3H, m, H-*sn*1, H-*sn*2), 3.56 (2H, t, *J* = 6.6 Hz, H-1′′), 2.20–1.32 (16H, m, H-5′, 7′, 8′, 11′, 12′, 13′, 14′, 16′, 17′), 1.70/1.67 (3H, s, Me-21′), 1.54 (2H, m, H-2′′), 1.25 (30H, m, H-3′′-17′′), 0.90 (3H, s, Me-22′), 0.88 (3H, t, *J* = 7.0 Hz, Me-18′′), 0.88 (3H, s, Me-24′), 0.81/0.80 (3H, d, *J* = 7.0 Hz, Me-23′); ^13^C-NMR (100 MHz, CDCl_3_, δ ppm): 154.6 (–O–CO–O–), 147.0 (C-19′), 143.2 (C-1′), 141.4/141.1 (C-10′), 140.2/140.1 (C-25′), 125.9 (C-3′), 120.0/119.7 (C-9′), 109.1/109.0 (C-20′), 108.6 (C-2′), 77.5/77.4 (C-*sn*2), 70.6 (C-1′′), 70.3 (C-4′), 66.2/66.1 (C-*sn*1), 61.8 (C-*sn*3), 44.2 (C-5′), 42.9 (C-15′), 42.4/42.3 (C-11′), 38.8 (C-14′), 37.3/37.2 (C-16′), 34.1/34.0 (C-6′), 32.4/32.3 (C-17′), 31.9 (C-16′′), 29.9–28.8 (C-2′′), 29.9–28.8 (C-4′′-15′′), 29.9–28.8 (C-7′, 13′), 26.0 (C-3′′), 22.8 (C-24′), 22.7 (C-8′, 12′), 22.6 (C-17′′), 22.2 (C-21′), 22.2 (C-22′), 15.6 (C-23′), 14.0 (C-18′′); EIHRMS: calcd. for C_46_H_78_O_6_ [M + Na]^+^: 749.5691, found: 749.5718.

### 4.30. Preparation of 1-O-[1,25-Epoxy-18-nor-ent-isodysidiola-1,3(25),9,19-tetraen-4R/S-yloxycarbonyl]-2-O-octadecyl-3-eicosapentaenoyl-sn-glycerol *(**42**)*

To a solution of **41** (18 mg, 0.025 mmol), DMAP (4 mg, 0.032 mmol) and EDAC (6 mg, 0.032 mmol) in dry CH_2_Cl_2_ (0.24 mL), EPA (8 mg, 0.026 mmol) was added under an argon atmosphere. After stirring at rt for 14 h, the reaction mixture was passed through a short silica gel column (CH_2_Cl_2_/EtOAc 9:1 as eluent), the solvent removed and the crude purified by column chromatography (Hex/EtOAc 99:1) providing **42** (16 mg, 63%). IR (film, cm^−1^): 2959, 2924, 2853, 1744, 1258; ^1^H-NMR (400 MHz, CDCl_3_, δ ppm): 7.42 (1H, broad s, H-25′), 7.34 (1H, broad s, H-1′), 6.39 (1H, s, H-2′), 5.79–5.76 (1H, m, H-4′), 5.40–5.30 (10H, m, =CH), 5.40–5.30 (1H, m, H-9′), 4.65/4.61 (2H, m, H-20′), 4.23–4.06 (2H, m, H-*sn*3), 4.23–4.06 (2H, m, H-*sn*1), 3.66 (1H, m, H-*sn*2), 3.50 (2H, t, *J* = 6.6 Hz, H-1′′), 2.84–2.80 (8H, m, =CCH_2_C=), 2.32 (2H, t, *J* = 7.5 Hz, H-2′′′), 2.13–2.06 (4H, m, H-4′′′, 19′′′), 2.04–1.40 (16H, m, H-5′, 7′, 8′, 11′, 12′, 13′, 14′, 16′, 17′), 1.82–1.79 (2H, m, H-3′′′), 1.69/1.66 (3H, s, Me-21′), 1.56 (2H, m, H-2′′), 1.25 (30H, m, H-3′′-17′′), 0.97 (3H, t, *J* = 7.6 Hz, H-20′′′), 0.89 (3H, s, Me-22′), 0.88 (3H, t, *J* = 7.0 Hz, Me-18′′), 0.88 (3H, s, Me-24′), 0.80 (3H, d, *J* = 6.8 Hz, Me-23′); ^13^C-NMR (50 MHz, CDCl_3_, δ ppm): 173.4 (C-1′′′), 154.7 (–O–CO–O–), 147.3 (C-19′), 143.5 (C-1′), 141.6/141.3 (C-10′), 140.4 (C-25′), 132.3 (C-18′′′), 129.1–127.2 (=CH) × 9, 126.2 (C-3′), 120.3/120.0 (C-9′), 109.4/109.3 (C-20′), 108.9 (C-2′), 75.2 (C-*sn*2), 71.0 (C-1′′), 70.5 (C-4′), 66.9/66.7 (C-*sn*3), 63.1 (C-*sn*1), 44.4 (C-5′), 43.1 (C-15′), 42.8/42.6 (C-11′), 39.1/38.9 (C-14′), 37.6/37.4 (C-16′), 34.4/34.3 (C-6′), 33.7 (C-2′′′), 32.7/32.6 (C-17′), 32.2 (C-16′′), 30.6 (C-7′), 30.1–29.2 (C-2′′), 30.1–29.2 (C-4′′-15′′), 30.1–29.2 (C-13′), 26.8–20.8 (3′′′, 4′′′, 7′′′, 10′′′, 13′′′, 16′′′, 19′′′, 17′′, 8′, 12′), 25.8 (C-3′′), 23.9 (C-24′), 22.9 (C-21′), 22.5 (C-22′), 15.9 (C-23′), 14.5 (C-20′′′), 14.3 (C-18′′); EIHRMS: calcd. for C_66_H_106_O_7_ [M + Na]^+^: 1033.7831, found: 1033.7866.

### 4.31. Preparation of 1-O-[25-Hydroxy-18-nor-ent-isodysidiola-2,9,19-trien-1,25-olide-4R/S-yloxycarbonyl]--2-O-octadecyl-3-eicosapentaenoyl-sn-glycerol *(**12**)*

Rose Bengal (2 mg) was added to a solution of **42** (16 mg, 0.016 mmol) and DIPEA (28 μL, 0.16 mmol) in dry CH_2_Cl_2_ (2 mL) at rt. Anhydrous oxygen was bubbled in for 5 min and, the solution placed under an oxygen atmosphere at −78 °C and irradiated with a 200 W lamp. After 4 h irradiation was stopped, the pink solution allowed to warm to rt, and saturated aqueous oxalic acid solution (2.5 mL) added. After a few minutes of vigorous stirring, the mixture was diluted with H_2_O (2 mL) and extracted with Et_2_O. The combined organic extracts were washed with H_2_O and dried over anhydrous Na_2_SO_4_. After filtration, the solvent was evaporated to give a residue which was purified by silica gel column chromatography to yield **12** (9 mg, 54%). IR (film, cm^−1^): 3402, 2959, 2924, 2853, 1751, 1256, 1136, 1126; ^1^H-NMR (400 MHz, CDCl_3_, δ ppm): 6.19/5.95 (1H, m, H-25′), 6.02/6.01 (1H, s, H-2′), 5.64–5.58 (1H, m, H-4′), 5.38–5.34 (10H, m, =CH), 5.38–5.34 (1H, m, H-9′), 4.67–4.63 (2H, m, H-20′), 4.34–3.98 (4H, m, H-*sn*1, H-*sn*3), 3.70–3.66 (1H, m, H-*sn*2), 3.53 (2H, t, *J* = 6.4 Hz, H-1′′), 2.85–2.78 (8H, m, =CCH_2_C=), 2.34 (2H, t, *J* = 7.2 Hz, H-2′′′), 2.13–2.04 (4H, m, H-4′′′, 19′′′), 2.00–1.49 (16H, m, H-5′, 7′, 8′, 11′, 12′, 13′, 14′, 16′, 17′), 1.84 (2H, m, H-3′′′), 1.70/1.69 (3H, s, Me-21′), 1.54 (2H, m, H-2′′), 1.25 (30H, m, H-3′′-17′′), 0.97 (3H, t, *J* = 7.6 Hz, H-20′′′), 0.91 (3H, s, Me-22′), 0.87 (3H, t, *J* = 6.8 Hz, Me-18′′), 0.87 (3H, s, Me-24′), 0.80 (3H, d, *J* = 6.9 Hz, Me-23′); ^13^C-NMR (100 MHz, CDCl_3_, δ ppm): 173.3 (C-1′′′), 169.0 (C-1′), 167.5 (C-3′), 154.2 (O–CO–O), 147.2 (C-19′), 141.4 (C-10′), 132.0 (C-18′′′), 128.9–127.0 (=CH) × 9, 120.0 (C-9′), 118.4 (C-2′), 109.2 (C-20′), 97.4 (C-25′), 76.5 (C-*sn*2), 70.8 (C-1′′), 70.8 (C-4′), 66.5 (C-*sn*1), 62.3 (C-*sn*3), 43.1 (C-5′), 42.8 (C-15′), 42.3 (C-11′), 38.4 (C-14′), 37.2 (C-16′), 34.6 (C-6′), 33.5 (C-2′′′), 32.4 (C-17′), 31.9 (C-16′′), 29.8–29.3 (C-2′′, 4′′-15′′), 28.9 (C-13′), 29.3 (C-7′), 26.5–20.5 (3′′′, 4′′′, 7′′′, 10′′′, 13′′′, 16′′′, 19′′′, 3′′), 22.8 (C-12′), 22.8 (C-8′), 22.7 (C-24′), 22.7 (C-21′), 22.6 (C-17′′), 22.5 (C-22′), 15.6 (C-23′), 14.2 (C-20′′′), 14.0 (C-18′′); EIHRMS: calcd. for C_66_H_106_O_9_ [M + Na]^+^: 1065.7729, found: 1065.7725.

### 4.32. Preparation of 1,25-Epoxy-18-nor-ent-isodysidiola-1,3(25),9,19-tetraen-4R/S-yl eicosapentaenoate *(**13**)*

To a solution of **1**/**2** (36 mg, 0.1 mmol), DMAP (16 mg, 0.13 mmol) and EDAC (25 mg, 0.13 mmol) in dry CH_2_Cl_2_ (1 mL), EPA (30 mg, 0.1 mmol) was added under an argon atmosphere. After stirring at rt for 20 h, the reaction mixture was passed through a short silica gel column ( CH_2_Cl_2_/EtOAc/ 9:1 as eluent), the solvent removed and the crude product purified by column chromatography to give **13** (40 mg, 63%). ^1^H-NMR (400 MHz, CDCl_3_, δ ppm): 7.38 (1H, s, H-25), 7.32 (1H, s, H-1), 6.35 (1H, s, H-2), 5.98/5.96 (1H, dd, *J* = 5.1, 3.4 Hz, H-4), 5.39–5.32 (10H, m, =CH), 5.39–5.32 (1H, m, H-9), 4.64 (1H, broad s, H_A_-20), 4.61 (1H, broad s, H_B_-20), 2.85–2.75 (8H, m, =CCH_2_C=), 2.25 (2H, t, *J* = 7.6 Hz, H-2′), 2.12–2.03 (4H, m, H-4′, 19′), 2.03–1.30 (16H, m, H-5, 7, 8, 11, 12, 13, 14, 16, 17), 1.87–1.78 (2H, m, H-3′), 1.70/1.67 (3H, s, Me-21), 0.97 (3H, t, *J* = 7.5 Hz, H-20′), 0.90 (3H, s, Me-22), 0.88 (3H, s, Me-24), 0.81/0.80 (3H, d, *J* = 6.9 Hz, Me-23); ^13^C-NMR (100 MHz, CDCl_3_, δ ppm): 172.8 (C-1′), 147.0 (C-19), 143.0 (C-1), 141.3 (C-10), 140.0 (C-25), 132.0 (C-18′), 129.0–127.0 (=CH) × 9, 126.7 (C-3), 119.9/119.8 (C-9), 109.0 (C-20), 108.8 (C-2), 65.4 (C-4), 44.0 (C-5), 42.9 (C-15), 42.4 (C-11), 38.9/38.8 (C-14), 37.2 (C-16), 34.2/34.1 (C-6), 34.0 (C-2′), 32.3 (C-17), 29.7 (C-7), 29.6 (C-13), 28.9–20.5 (3′, 4′, 7′, 10′, 13′, 16′, 19′), 22.8 (C-24), 22.7 (C-8, 12), 22.3 (C-21), 22.3 (C-22), 15.6 (C-23), 14.2 (C-20′); EIHRMS: calcd. for C_44_H_64_O_3_ [M + Na]^+^: 663.4748, found: 663.4747.

### 4.33. Preparation of 4-Eicosapentaenoyl-25-hydroxy-18-nor-ent-isodysidiola-2,9,19-trien-1,25-olide *(**14**)*

Rose Bengal (2 mg) was added to a solution of **13** (18 mg, 0.028 mmol) and DIPEA (36 mg, 0.28 mmol) in dry CH_2_Cl_2_ (4 mL) at rt. Anhydrous oxygen was bubbled in for 5 min., the solution placed under an oxygen atmosphere at −78 °C and irradiated with a 200 W lamp. After 4 h irradiation was stopped, the pink solution allowed to warm to rt, and saturated aqueous oxalic acid solution (3 mL) added. After a few minutes of vigorous stirring, the mixture was diluted with H_2_O (3 mL) and extracted with Et_2_O. The combined organic extracts were washed with H_2_O and dried over anhydrous Na_2_SO_4_. After filtration, the solvent was evaporated to give a residue which was purified by silica gel column chromatography (Hex/EtOAc 95:5) to yield **14** (10 mg, 54%). IR (film, cm^−1^): 3389, 2961, 2926, 2872, 1744, 1142; ^1^H-NMR (400 MHz, CDCl_3_, δ ppm): 6.19 (1H, m, H-25)/5.96 (1H, s, H-25), 5.99/5.93 (1H, s, H-2), 5.58 (1H, d, *J* = 9.0 Hz, H-4), 5.44–5.32 (10H, m, =CH), 5.44–5.32 (1H, m, H-9), 4.66 (1H, broad s, H_A_-20), 4.61 (1H, broad s, H_B_-20), 2.86–2.78 (8H, m, =CCH_2_C=), 2.37–2.30 (2H, m, H-2′), 2.12–2.06 (4H, m, H-4′, 19′), 2.06–1.30 (16H, m, H-5, 7, 8, 11, 12, 13, 14, 16, 17), 1.89–1.83 (2H, m, H-3′), 1.70 (3H, s, Me-21), 0.97 (3H, t, *J* = 7.5 Hz, H-20′), 0.92 (3H, s, Me-22), 0.89 (3H, s, Me-24), 0.81 (3H, d, *J* = 6.9 Hz, Me-23); ^13^C-NMR (50 MHz, CDCl_3_, δ ppm): 173.3 (C-1′), 169.5 (C-1), 168.7 (C-3), 147.3 (C-19), 141.4 (C-10), 132.3 (C-18′), 129.5–127.2 (=CH) × 9, 120.1 (C-9), 118.3 (C-2), 109.5 (C-20), 97.9 (C-25), 67.2 (C-4), 43.1 (C-15), 42.9 (C-11), 42.8 (C-5), 39.0/38.6 (C-14), 37.6 (C-16), 34.9/34.6 (C-6), 33.9 (C-2′), 32.7 (C-17), 31.6 (C-7), 28.9 (C-13), 26.7–20.8 (3′, 4′, 7′, 10′, 13′, 16′, 19′, 8, 12), 23.0 (C-24), 22.4 (C-21), 22.4 (C-22), 15.8 (C-23), 14.5 (C-20′); EIHRMS: calcd. for C_44_H_64_O_5_ [M + Na]^+^: 695.4646, found: 695.4669.

## 5. Conclusions

In summary, we have synthesized several bioconjugate compounds combining sesterterpenoids, alkyl glycerol chains and PUFAs. The *in vitro* antitumour activity of these compounds was studied against the HeLa and MCF-7 tumour cell lines. From the results reported here, several conclusions could be deduced: (a) the change of a furan for a γ-hydroxybutenolide unit increases the biological antitumour activity; (b) bioconjugation of γ-hydroxybutenolide sesterterpenes with glycerol derivatives and PUFAs increase the activity with respect to the sesterterpenoids in the edelfosine range; (c) simple bioconjugates of a sesterterpenoid and EPA, as γ-hydroxybutenolide **14**, show the best biological activity for the tumour cell lines tested. In this respect, compounds **11** and **12** are in the range of edelfosine for HeLa cells and slightly better for MCF-7 cells. The remarkable activity of compound **14** makes of it a very interesting molecule for further studies and shows the synergy of bioconjugation of sesterterpenolides and PUFAs. Additional experiments are needed to establish the scope and limitations of this behaviour.

## References

[1] Nilo A., Allan M., Brogioni B., Proietti D., Cattaneo V., Crotti S., Sokup S., Zhai H., Margarit I., Berti F. (2014). Tyrosine-Directed Conjugation of Large Glycans to Proteins via Copper-Free Click Chemistry. Bioconjugate Chem..

[2] Shenvi S., Kiran K.R., Kumar K., Diwakar L., Reddy G.C. (2015). Synthesis and biological evaluation of boswellic acid-NSAID hybrid molecules as anti-inflammatory and anti-arthritic agents. Eur. J. Med. Chem..

[3] Romero-Hernández L.L., Merino-Montiel P., Montiel-Smith S., Meza-Reyes S., Vega-Báez J.L., Abasolo I., Schwartz S., López Ó., Fernández-Bolaños J.G. (2015). Diosgenin-based thio(seleno)ureas and triazolyl glycoconjugates as hybrid drugs. Antioxidant and antiproliferative profile. Eur. J. Med. Chem..

[4] Zamudio-Vazquez R., Ivanova S., Moreno M., Hernandez-Alvarez M.I., Giralt E., Bidon-Chanal A., Zorzano A., Albericio F., Tulla-Puche J. (2015). A new quinoxaline-containing peptide induces apoptosis in cancer cells by autophagy modulation. Chem. Sci..

[5] Lampkowski J.S., Villa J.K., Young T.S., Young D.D. (2015). Development and Optimization of Glaser—Hay Bioconjugations. Angew. Chem. Int. Ed..

[6] Raouane M., Desmaële D., Urbinati G., Massaad-Massade L., Couvreur P. (2012). Lipid Conjugated Oligonucleotides: A Useful Strategy for Delivery. Bioconjugate Chem..

[7] Vallinayagam R., Weber J., Neier R. (2008). Novel Bioconjugates of Aminolevulinic Acid with Vitamins. Org. Lett..

[8] Ainge G.D., Compton B.J., Hayman C.M., Martin W.J., Toms S.M., Larsen D.S., Harper J.L., Painter G.F. (2011). Chemical Synthesis and Immunosuppressive Activity of Dipalmitoyl Phosphatidylinositol Hexamannoside. J. Org. Chem..

[9] Bradley M.O., Webb N.L., Anthony F.H., Devanesan P., Witman P.A., Hemamalini S., Chander M.C., Baker S.D., He L., Horwitz S.B. (2001). Tumor Targeting by Covalent Conjugation of a Natural Fatty Acid to Paclitaxel. Clin. Cancer Res..

[10] Kuznetsova L., Chen J., Sun L., Wu X., Pepe A., Veith J.M., Pera P., Bernacki R.J., Ojima I. (2006). Syntheses and evaluation of novel fatty acid-second-generation taxoid conjugates as promising anticancer agents. Bioorg. Med. Chem. Lett..

[11] Pedersen P.J., Christensen M.S., Ruysschaert T., Linderoth L., Andresen T.L., Melander F., Mouritsen O.G., Madsen R., Clausen M.H. (2009). Synthesis and Biophysical Characterization of Chlorambucil Anticancer Ether Lipid Prodrugs. J. Med. Chem..

[12] Linderoth L., Peters G.H., Madsen R., Andresen T.L. (2009). Drug Delivery by an Enzyme-Mediated Cyclization of a Lipid Prodrug with Unique Bilayer-Formation Properties. Angew. Chem. Int. Ed..

[13] Pedersen P.J., Adolph S.K., Subramanian A.K., Arouri A., Andresen T.L., Mouritsen O.G., Madsen R., Madsen M.W., Peters G.H., Clausen M.H. (2010). Liposomal Formulation of Retinoids Designed for Enzyme Triggered Release. J. Med. Chem..

[14] Christensen M.S., Pedersen P.J., Andresen T.L., Madsen R., Clausen M.H. (2010). Isomerization of all-(*E*)-Retinoic Acid Mediated by Carbodiimide Activation—Synthesis of ATRA Ether Lipid Conjugates. Eur. J. Org. Chem..

[15] Pedersen P.J., Adolph S.K., Andresen T.L., Madsen M.W., Madsen R., Clausen M.H. (2010). Prostaglandin phospholipid conjugates with unusual biophysical and cytotoxic properties. Bioorg. Med. Chem. Lett..

[16] Jung M.E., Berliner J.A., Koroniak L., Gugiu B.G., Watson A.D. (2008). Improved Synthesis of the Epoxy Isoprostane Phospholipid PEIPC and its Reactivity with Amines. Org. Lett..

[17] Pedersen P.J., Viart H.M.F., Melander F., Andresen T.L., Madsen R., Clausen M.H. (2012). Synthesis of tocopheryl succinate phospholipid conjugates and monitoring of phospholipase A2 activity. Bioorg. Med. Chem..

[18] Huang Z., Szoka F.C. (2008). Sterol-Modified Phospholipids: Cholesterol and Phospholipid Chimeras with Improved Biomembrane Properties. J. Am. Chem. Soc..

[19] Huang Z., Jaafari M.R., Szoka F.C. (2009). Disterolphospholipids: Nonexchangeable Lipids and Their Application to Liposomal Drug Delivery. Angew. Chem. Int. Ed..

[20] Magnusson C.D., Gudmundsdottir A.V., Haraldsson G.G. (2011). Chemoenzymatic synthesis of a focused library of enantiopure structured 1-*O*-alkyl-2,3-diacyl-sn-glycerol type ether lipids. Tetrahedron.

[21] Gunasekera S.P., Mccarthy P.J., Kelly-Borges M., Lobkovsky E., Clardy J. (1996). Dysidiolide: A Novel Protein Phosphatase Inhibitor from the Caribbean Sponge Dysidea etheria de Laubenfels. J. Am. Chem. Soc..

[22] Corey E.J., Roberts B.E. (1997). Total Synthesis of Dysidiolide. J. Am. Chem. Soc..

[23] Brohm D., Philippe N., Metzger S., Bhargava A., Müller O., Lieb F., Waldmann H. (2002). Solid-Phase Synthesis of Dysidiolide-Derived Protein Phosphatase Inhibitors. J. Am. Chem. Soc..

[24] Brohm D., Metzger S., Bhargava A., Müller O., Lieb F., Waldmann H. (2002). Natural Products Are Biologically Validated Starting Points in Structural Space for Compound Library Development: Solid-Phase Synthesis of Dysidiolide-Derived Phosphatase Inhibitors. Angew. Chem. Int. Ed..

[25] Gajate C., Mollinedo F. (2014). Lipid Rafts, Endoplasmic Reticulum and Mitochondria in the Antitumor Action of the Alkylphospholipid Analog EdelfosineLipid Rafts, Endoplasmic Reticulum and Mitochondria in the Antitumor Action of the Alkylphospholipid Analog Edelfosine. Anticancer Agents Med. Chem..

[26] Gajate C., Mollinedo F. (2002). Biological Activities, Mechanisms of Action and Biomedical Prospect of the Antitumor Ether Phospholipid ET-18-OCH_3_ (Edelfosine), A Proapoptotic Agent in Tumor Cells. Curr. Drug. Metab..

[27] Lohmeyer M., Bittman R. (1994). Antitumor ether lipids and alkylphosphocholines. Drugs Fut..

[28] Reis-Sobreiro M., Roue G., Moros A., Gajate C., de la Iglesia-Vicente J., Colomer D., Mollinedo F. (2013). Lipid raft-mediated Akt signaling as a therapeutic target in mantle cell lymphoma. Blood Cancer J..

[29] Gajate C., Matos-Da-Silva M., Dakir E.L.H., Fonteriz R.I., Alvarez J., Mollinedo F. (2012). Antitumor alkyl-lysophospholipid analog edelfosine induces apoptosis in pancreatic cancer by targeting endoplasmic reticulum. Oncogene.

[30] Gajate C., del Canto-Jañez E., Acuña A.U., Amat-Guerri F., Geijo E., Santos-Beneit A.M., Veldman R.J., Mollinedo F. (2004). Intracellular Triggering of Fas Aggregation and Recruitment of Apoptotic Molecules into Fas-enriched Rafts in Selective Tumor Cell Apoptosis. J. Exp. Med..

[31] Mollinedo F., Martinezdalmau R., Modolell M. (1993). Early and Selective Induction of Apoptosis in Human Leukemic Cells by the Alkyl-Lysophospholipid ET-18-OCH_3_. Biochem. Biophys. Res. Commun..

[32] Gunstone F.D. (2004). The Chemistry of Oils and Fats. Sources, Composition, Properties and Uses.

[33] Wigmore S.J., Ross J.A., Falconer J.S., Plester C.E., Tisdale M.J., Carter D.C., Fearon K.C. (1996). The effect of polyunsaturated fatty acids on the progress of cachexia in patients with pancreatic cancer. Nutrition.

[34] Jahn U., Galano J.-M., Durand T. (2008). Beyond prostaglandins-chemistry and biology of cyclic oxygenated metabolites formed by free-radical pathways from polyunsaturated fatty acids. Angew. Chem. Int. Ed..

[35] Magnusson C.D., Haraldsson G.G. (2010). Chemoenzymatic synthesis of symmetrically structured triacylglycerols possessing short-chain fatty acids. Tetrahedron.

[36] Heird W.C., Lapillonne A. (2005). The role of essential fatty acids in development. Annu. Rev. Nutr..

[37] Hardman W.E. (2002). Omega-3 fatty acids to augment cancer therapy. J. Nutr..

[38] Hawkins R.A., Sangster K., Arends M.J. (1998). Apoptotic death of pancreatic cancer cells induced by polyunsaturated fatty acids varies with double bond number and involves an oxidative mechanism. J. Pathol..

[39] Sauer L.A., Dauchy R.T., Blask D.E. (2000). Mechanism for the antitumor and anticachectic effects of n-3 fatty acids. Cancer Res..

[40] Sauer L.A., Dauchy R.T. (1992). The effect of omega-6 and omega-3 fatty acids on 3*H*-thymidine incorporation in hepatoma 7288CTC perfused *in situ*. Br. J. Cancer.

[41] Grammatikos S.I., Subbaiah P.V., Victor T.A., Miller W.M. (1994). N-3 and n-6 fatty acid processing and growth effects in neoplastic and non-cancerous human mammary epithelial cell lines. Br. J. Cancer.

[42] Cao W.Q., Ma Z.F., Rasenick M.M., Yeh S.Y., Yu J.Z. (2012). N-3 poly-unsaturated fatty acids shift estrogen signaling to inhibit human breast cancer cell growth. PLoS ONE.

[43] Gudmundsdottir A.V., Hansen K.-A., Magnusson C.D., Haraldsson G.G. (2015). Synthesis of reversed structured triacylglycerols possessing EPA and DHA at their terminal positions. Tetrahedron.

[44] Mollinedo F., Gajate C., Martin-Santamaria S., Gago F. (2004). ET-18-OCH_3_ (edelfosine): A selective antitumour lipid targeting apoptosis through intracellular activation of fas/CD95 death receptor. Curr. Med. Chem..

[45] Mollinedo F., Fernández-Luna J.L., Gajate C., Martín-Martín B., Benito A., Martínez-Dalmau R., Modolell M. (1997). Selective Induction of Apoptosis in Cancer Cells by the Ether Lipid ET-18-OCH_3_ (Edelfosine): Molecular Structure Requirements, Cellular Uptake, and Protection by Bcl-2 and Bcl-XL. Cancer Res..

[46] Marcos I.S., Escola M.A., Moro R.F., Basabe P., Diez D., Sanz F., Mollinedo F., de la Iglesia-Vicente J., Sierra B.G., Urones J.G. (2007). Synthesis of novel antitumoral analogues of dysidiolide from *ent*-halimic acid. Bioorg. Med. Chem..

[47] Samadder P., Bittman R., Byun H.S., Arthur G. (2004). Synthesis and Use of Novel Ether Phospholipid Enantiomers To Probe the Molecular Basis of the Antitumor Effects of Alkyllysophospholipids: Correlation of Differential Activation of c-Jun NH2-Terminal Protein Kinase with Antiproliferative Effects in Neuronal Tumor Cells. J. Med. Chem..

[48] Nagashima N., Ohno M. (1987). An efficient *O*-monoalkylation of dimethyl l-tartrate via *O*-stannylene acetal with alkyl halides in the presence of cesium fluoride. Chem. Lett..

[49] Byun H.S., Kumar E.R., Bittman R. (1994). Enantioselective Syntheses of 1-*O*-Benzyl-sn-glycerol and 1-*O*-Hexadecyl-2-*O*-methyl-sn-glycerol via Asymmetric Dihydroxylation of Allyl 4-Methoxyphenyl Ether. Use of AD-Mix Supplemented with Potassium Persulfate. J. Org. Chem..

[50] Johansson R., Samuelsson B. (1984). Regioselective reductive ring-opening of 4-methoxybenzylidene acetals of hexopyranosides. Access to a novel protecting-group strategy. Part 1. J. Chem. Soc. Perkin Trans..

[51] Horita K., Yoshioka T., Tanaka T., Oikawa Y., Yonemitsu O. (1986). On the selectivity of deprotection of benzyl, MPM (4-methoxybenzyl) and DMPM (3,4-dimethoxybenzyl) protecting groups for hydroxy functions. Tetrahedron.

[52] Kernan M.R., Faulkner D.J. (1988). Regioselective oxidation of 3-alkylfurans to 3-alkyl-4-hydroxybutenolides. J. Org. Chem..

[53] Harbison G.S., Griffin R.G. (1984). Improved method for the synthesis of phosphatidylcholines. J. Lipid Res..

[54] Langlois N., Legeay J.-C., Retailleau P. (2008). C-(4-methoxybenzyloxymethyl)-*N*-methylnitrone cycloaddition to highly functionalized pyrrolinone: A regio- and stereoselective approach to new omuralide-salinosporamide a hybrids. Eur. J. Org. Chem..

[55] Greene T., Wuts P.G.M. (1999). Protective Groups in Organic Shynthesis.

[56] Ichihara A., Ubukata M., Sakamura S. (1977). Stereoselective synthesis of (±)-palitantin. Tetrahedron Lett..

[57] David-Cordonnier M.H., Gajate C., Olmea O., Laine W., de la Iglesia-Vicente J., Perez C., Cuevas C., Otero G., Manzanares I., Bailly C. (2005). DNA and Non-DNA Targets in the Mechanism of Action of the Antitumor Drug Trabectedin. Chem. Biol..

[58] Marcos I.S., Pedrero A.B., Sexmero M.J., Diez D., Basabe P., García N., Moro R.F., Broughton H.B., Mollinedo F., Urones J.G. (2003). Synthesis of Bioactive Sesterterpenolides from *ent*-Halimic Acid. 15-Epi-ent-cladocoran A and B. J. Org. Chem..

[59] Marcos I.S., Pedrero A.B., Sexmero M.J., Diez D., Basabe P., Hernández F.A., Urones J.G. (2003). Synthesis and absolute configuration of three natural ent-halimanolides with biological activity. Tetrahedron Lett..

